# Preoperative Prediction of Lymph Node Metastasis in Colorectal Cancer Using Routinely Collected Clinical Data: Multicenter Retrospective Machine Learning Model Development and External Validation Study

**DOI:** 10.2196/101162

**Published:** 2026-10-07

**Authors:** Gehui Xu, Xiaohe Sun, Jiaxin Jiang, Jin Sun, Liu Li, Haibo Cheng, Yuekun Zhu, Haiyi Liu

**Affiliations:** 1 First Clinical Medical College, Nanjing University of Chinese Medicine, 138 Xianlin Rd, Qixia District, Nanjing, 210023, China, 86 25-85811079, 86 25-85811078; 2 Department of General Surgery, First Affiliated Hospital of Harbin Medical University, Harbin, Heilongjiang, China; 3 Department of Colorectal Surgery, Shanxi Provincial Cancer Hospital, Taiyuan, Shanxi, China

**Keywords:** colorectal cancer, lymph node metastasis, machine learning, random forest, preoperative prediction, external validation, SHAP, risk stratification, shapley additive explanations

## Abstract

**Background:**

Preoperative nodal assessment in colorectal cancer (CRC) remains imperfect, and uncertainty regarding pathological lymph node metastasis (LNM) may persist despite conventional imaging. Machine learning using routinely available preoperative information may provide complementary patient-level risk estimates without requiring radiomic feature extraction or molecular testing.

**Objective:**

This study aimed to develop and externally validate machine learning models for preoperative estimation of pathologically confirmed LNM risk in patients with newly diagnosed CRC undergoing direct radical surgery without preoperative anticancer treatment, and to assess whether multivariable prediction adds information beyond clinical tumor stage (cT) alone.

**Methods:**

This multicenter retrospective study included 2725 patients from 2 hospitals. The internal cohort (n=1074) was split into training (n=753) and internal test (n=321) sets, while 1651 patients from a second center formed the external validation cohort. Candidate predictors were routinely available preoperative clinical, laboratory, tumor-marker, imaging-based, and endoscopic biopsy variables. Predictors were selected using univariable and multivariable logistic regression (LR). Seven algorithms were evaluated: random forest (RF), support vector machine (SVM), LR, decision tree (DT), extreme gradient boosting (XGBoost), naive Bayes (NB), and light gradient boosting machine (LightGBM). Performance was assessed using discrimination, threshold-dependent classification metrics, calibration, and decision curve analysis (DCA). Shapley additive explanations (SHAP) were used for interpretation. Sensitivity analyses compared the selected predictors with all candidate predictors and cT alone.

**Results:**

Six predictors were retained: BMI, preoperative carcinoembryonic antigen, primary tumor site, cT, histological type, and tumor differentiation. RF was selected based on internal test performance. The area under the receiver operating characteristic curve (AUROC) was 0.803 (95% CI 0.756‐0.849) in the internal test set and 0.776 (95% CI 0.755‐0.797) in external validation. In external validation, accuracy, sensitivity, specificity, positive predictive value (PPV), and negative predictive value (NPV) were 0.687, 0.677, 0.693, 0.569, and 0.781, respectively. The Brier score was 0.190, and the calibration slope was 1.061. cT contributed most in SHAP analysis; however, the 6-predictor RF model showed significantly higher discrimination than cT alone in both the internal test set (AUROC 0.803 vs 0.712; ΔAUROC=0.091; *P*<.001) and external validation cohort (AUROC 0.776 vs 0.707; ΔAUROC=0.069; *P*<.001). Discrimination did not differ significantly between the 6-predictor and all-predictor RF models.

**Conclusions:**

We developed and externally validated a 6-predictor RF model for preoperative LNM risk estimation in patients with newly diagnosed CRC undergoing radical resection with regional lymph node dissection without preoperative anticancer treatment. The model showed moderate external discrimination and provided predictive information beyond cT alone. It is intended for research-stage adjunctive risk stratification and should not replace imaging, pathological evaluation, or clinical judgment. Prospective validation in broader populations and clinical settings is required before implementation.

## Introduction

Colorectal cancer (CRC) remains a major global health burden. According to Global Cancer Observatory (GLOBOCAN) 2024, more than 2.0 million new CRC cases and approximately 918,000 CRC-related deaths occurred worldwide in 2024, making CRC the third most commonly diagnosed cancer and the second leading cause of cancer death globally [1]. Regional lymph node metastasis (LNM) is a key component of CRC staging and an important prognostic factor [2]. Accurate preoperative assessment of nodal involvement is therefore important for defining disease extent and stratifying patient risk before surgery.

Conventional imaging is central to preoperative CRC staging and provides essential information on local tumor extent. However, radiological assessment of nodal involvement remains challenging because benign and metastatic lymph nodes may overlap in size and morphological appearance. A recent systematic review and meta-analysis found that size-based preoperative computed tomography (CT) achieved only moderate diagnostic performance for LNM in colon cancer [3]. Similarly, an updated review of rectal magnetic resonance imaging (MRI) identified nodal staging as a persistent challenge, with size and morphological criteria subject to substantial overlap and staging pitfalls [4]. Thus, although imaging provides indispensable anatomical information, uncertainty regarding pathological nodal status may remain before surgery. Integrating routinely available clinical, laboratory, and biopsy-derived information may therefore provide a more complete patient-level estimate of LNM risk.

Machine learning has increasingly been used to combine clinical, imaging, and pathological information for LNM prediction in CRC. However, existing models differ substantially in the populations studied, predictors included, and data modalities used, ranging from conventional clinical variables to radiomics and digital pathology, which complicates comparison across studies [5]. Models using high-dimensional imaging or pathological features may capture additional predictive information but also require more complex feature extraction and interpretation. Importantly, model performance observed during development may not be maintained in independent populations, and recent CRC-specific reviews have identified limited external validation as an important source of uncertainty in reported performance [6,7]. These issues are particularly relevant to preoperative LNM prediction, for which a useful model should provide reliable risk estimates using information that can be obtained consistently before surgery.

Accordingly, we aimed to develop and externally validate machine learning models for preoperative estimation of pathologically confirmed LNM risk in patients with newly diagnosed CRC undergoing direct radical surgery without preoperative anticancer treatment. Predictors were restricted to routinely available preoperative information, including clinical characteristics, laboratory and tumor-marker measurements, imaging-based clinical assessment, and endoscopic biopsy findings. We compared multiple machine learning algorithms, evaluated the selected model in an independent external cohort, and used model interpretation to characterize predictor contributions. We also assessed whether the multivariable model provided predictive information beyond clinical T stage alone. The intended use was adjunctive patient-level LNM risk stratification within the direct-surgery pathway.

## Methods

### Study Design, Setting, and Participants

This was a multicenter retrospective study for the development and external validation of a clinical prediction model. After ethics approval was obtained, investigators retrospectively collected data from patients with newly diagnosed CRC who underwent radical resection at the First Affiliated Hospital of Harbin Medical University or Shanxi Province Cancer Hospital between July 2023 and December 2025.

The inclusion criteria were as follows: (1) age ≥18 years; (2) a new diagnosis of CRC confirmed by postoperative pathology; (3) direct treatment with radical resection and regional lymph node dissection; and (4) ascertainable postoperative pathological regional LNM status. Patients managed with endoscopic resection, local excision, nonoperative treatment, or preoperative anticancer therapy were not eligible for the direct-surgery cohort. The exclusion criteria were as follows: (1) preoperative radiotherapy, chemotherapy, targeted therapy, immunotherapy, or other anticancer treatment; and (2) synchronous or metachronous malignant tumors other than CRC.

The sample size was determined by all consecutive eligible cases available during the prespecified study period, and no a priori sample size estimation was performed.

The First Affiliated Hospital of Harbin Medical University constituted the internal development cohort. Stratified random sampling based on LNM status was used to split the cohort into training and internal test sets at a 7:3 ratio. The random seed was fixed at one to ensure reproducibility. Shanxi Province Cancer Hospital constituted the independent external validation cohort.

### Outcome Definition and Candidate Predictors

The primary outcome was regional LNM, confirmed by the postoperative pathological report. Patients were then classified as LNM-positive or LNM-negative according to the final pathological findings.

Candidate predictors were prespecified before model construction and were all obtainable through routine preoperative clinical assessment.

The candidate predictors included the following categories: Demographic and general clinical variables were age, sex, ethnicity, residence, smoking history, drinking history, family history, comorbidities, and BMI. Laboratory indices were neutrophil percentage, systemic immune-inflammation index (SII), and albumin-to-globulin ratio (AGR). Tumor markers were preoperative carcinoembryonic antigen (pCEA) and preoperative carbohydrate antigen 19‐9 (pCA19-9). Tumor-related variables were clinical T stage, bowel obstruction, primary tumor site, maximum tumor diameter, gross tumor type, histological type, and tumor differentiation. Comorbidities were recorded as a binary variable (yes or no). Patients with a documented history of hypertension, diabetes mellitus, or coronary heart disease were classified as having comorbidities; patients without any of these conditions were classified as having no comorbidities.

Laboratory indicators were obtained from the most recent preoperative test results, and values measured within 1 week before surgery were used whenever available. SII was calculated as platelet count×absolute neutrophil count/absolute lymphocyte count. AGR was calculated as serum albumin/globulin, where globulin=total protein−albumin. Tumor differentiation and histological type were obtained from preoperative endoscopic biopsy pathology reports, whereas maximum tumor diameter and clinical T stage were obtained from preoperative imaging and/or endoscopic evaluation.

### Missing Data Handling

Before data preprocessing, the number and proportion of missing values for each candidate predictor were calculated separately in the training, internal test, and external validation sets. Patients with missing postoperative pathological LNM status were excluded before cohort formation, and the outcome was not imputed. The internal development cohort was divided into the training and internal test sets before missing-data handling, and the external validation cohort remained independent throughout. For limited missingness in continuous predictors, multiple imputation by chained equations was performed with the R package *mice* (Stef van Buuren and Karin Groothuis-Oudshoorn), generating 3 imputed data sets with a maximum of 50 iterations per chain and a random seed of 123. Missing categorical predictor values were imputed using the training-set mode. The imputation model and derived preprocessing rules were estimated from the training data and then applied without refitting to the internal test and external validation sets. Variable-level missingness before imputation is reported in Table S2 in Multimedia Appendix 1; because missingness was summarized by variable, the same patient could contribute to more than one missing-value count.

### Biopsy-Resection Agreement Analysis

Among patients with both preoperative endoscopic biopsy and postoperative resection specimen pathology results, the agreement in histological type and tumor differentiation was evaluated. This analysis included only patients with nonmissing preoperative and postoperative results for the corresponding pathological indicator, and no imputed values were used.

Histological type was uniformly classified as adenocarcinoma and special histological type, with the latter including mucinous adenocarcinoma and signet-ring cell carcinoma. Agreement was evaluated using a cross-classification table, the exact agreement rate, the proportion of special histological type missed on preoperative biopsy, the positive predictive value (PPV) of a preoperative diagnosis of special histological type, and the unweighted Cohen κ coefficient with its 95% CI. A missed special histological type was defined as a preoperative biopsy diagnosis of adenocarcinoma with a postoperative resection specimen diagnosis of special histological type, and its proportion was calculated with patients confirmed as having special histological type postoperatively as the denominator. Tumor differentiation was uniformly classified as G1, G2, and G3, G4. Concordance was assessed using cross-classification tables, the exact agreement rate, the proportion with underestimated grade, the proportion with overestimated grade, and the linearly weighted Cohen κ coefficient with its 95% CI. Underestimated grade was defined as a pathologic grade on preoperative biopsy that was lower than the pathologic grade on the postoperative resection specimen, indicating that the biopsy suggested a more favorable grade; overestimated grade was defined as a pathologic grade on preoperative biopsy that was higher than the pathologic grade on the postoperative resection specimen. The 95% CIs for all percentages were calculated using the Wilson method, and the 95% CI for the Cohen κ coefficient was estimated by 20,000 multinomial bootstrap resamples. Detailed results are presented in Table S3 in Multimedia Appendix 1.

### Predictor Preprocessing and Encoding

After missing-data handling, predictors were encoded according to their variable type before model development. BMI and pCEA were retained as continuous variables without standardization. Primary tumor site was one-hot encoded, with right-sided colon as the reference category and separate indicator variables for left-sided colon and rectum. Histological type was binary encoded as adenocarcinoma=0 and mucinous adenocarcinoma or signet-ring cell carcinoma=1. Clinical Tumor stage (cT) and tumor differentiation were encoded as ordinal integers ([clinical Tumor1 stage] cT1=0, [clinical Tumor2 stage] cT2=1, [clinical Tumor3 stage] cT3=2, [clinical Tumor4 stage] cT4=3; G1=0, G2=1, and G3 or G4=2, respectively).

The same preprocessing and encoding scheme was applied across all candidate machine learning models. The preprocessing rules were defined using the training data and then applied unchanged to the internal test and external validation sets. Detailed predictor preprocessing and encoding rules are provided in Table S5 in Multimedia Appendix 1.

### Predictor Selection

Predictor selection was performed exclusively in the training set to avoid information leakage. Prespecified candidate predictors were first evaluated using univariable binary logistic regression (LR). Variables associated with LNM at *P*<.05 were subsequently entered into a multivariable binary LR model. Variables remaining statistically significant at *P*<.05 in the multivariable analysis were retained as the final predictor set for subsequent machine learning model development. Associations were reported as odds ratios (ORs) with 95% CIs. The internal test and external validation sets were not used for predictor selection.

### Model Development and Hyperparameter Tuning

Using the predictors retained through the prespecified selection procedure, 7 machine learning models were developed: random forest (RF), support vector machine (SVM), LR, decision tree (DT), extreme gradient boosting (XGBoost), naive Bayes (NB), and light gradient-boosting machine (LightGBM). All model development and hyperparameter tuning were performed exclusively using the training set. In the training set, LNM was present in 278 of 753 (36.9%) patients. RF therefore used class_weight=balanced to account for the moderate outcome imbalance. Final hyperparameter settings are provided in Table S5 in Multimedia Appendix 1.

For RF, SVM, DT, XGBoost, and LightGBM, hyperparameters were optimized using grid search with 3-fold cross-validation within the training set, with the area under the receiver operating characteristic curve (AUROC) as the optimization metric. LR and NB were fitted without hyperparameter tuning. The final hyperparameter settings for all candidate models are provided in Table S5 in Multimedia Appendix 1.

### Model Performance Evaluation and Validation

Model performance was evaluated in the training, internal test, and independent external validation sets. Discrimination was assessed using receiver operating characteristic (ROC) curves and the AUROC with 95% CIs. Classification performance was evaluated using accuracy, sensitivity, specificity, *F*_1_ score, PPV, and negative predictive value (NPV).

For each model, an evaluation operating point was determined by maximizing the Youden index in the training set and was then applied unchanged to the internal test and external validation sets to calculate threshold-dependent classification metrics. No operating-point reoptimization was performed using the internal test or external validation data. These training-derived operating points were used for model evaluation and were not considered validated clinical decision cutoffs.

Calibration was assessed using calibration curves, the Brier score, calibration intercept, and calibration slope. Clinical utility was explored using decision curve analysis (DCA) by estimating net benefit across a prespecified threshold-probability display interval of 0.05‐0.80. The final model was selected based on prespecified overall performance in the internal test set, considering discrimination, classification performance, calibration, clinical utility, and the balance of sensitivity, specificity, and *F*_1_ score. After model selection, the selected model was evaluated in the independent external validation cohort without further fitting, hyperparameter tuning, or threshold optimization. The DCA interval was descriptive and was not treated as a validated clinical decision threshold.

After selection of the final model, its AUROC was compared pairwise with those of the other candidate models in the internal test and external validation sets using the DeLong test. To account for multiple comparisons, *P* values were adjusted separately within each data set using the Holm method.

### Model Interpretability

Shapley additive explanations (SHAP) analysis was performed for the model selected according to the prespecified evaluation framework to characterize the contribution of individual predictors to model output. Global feature importance was quantified using the mean absolute SHAP value for each encoded feature. A SHAP summary plot was used to visualize both the magnitude and direction of predictor contributions across patients.

Patient-level interpretation was performed using an individual SHAP explanation to illustrate how multiple predictors jointly contributed to a single prediction. SHAP dependence plots were additionally generated to examine the relationship between predictor values and their contributions to model output. For primary tumor site, the 2 dummy variables representing left-sided colon and rectum were interpreted separately, with right-sided colon serving as the reference category; the dummy-variable values were not interpreted as an ordinal risk gradient.

### Sensitivity Analyses

Sensitivity analyses were performed to examine the robustness of the predictor-selection strategy and the incremental predictive value of the selected predictor set beyond clinical T stage alone. Using the algorithm selected as the final model, 3 models were constructed: a model based on the predictor set retained through the prespecified selection procedure, a model incorporating all prespecified candidate predictors, and a model using clinical T stage alone.

All models were developed using the training set and evaluated in the internal test and external validation sets. Their performance was compared in terms of discrimination, threshold-dependent classification performance, calibration, and clinical utility. Pairwise AUROC comparisons between the 6-predictor model and the 2 comparator models were performed using the DeLong test. Detailed sensitivity-analysis results are presented in Table S10 and Figure S4 in Multimedia Appendix 1.

### Statistical Analysis and Software

Two-sided tests were used for all analyses. Statistical significance was defined at α=.05. We assessed normality of continuous variables with the Shapiro-Wilk test. Normally distributed data are presented as mean (SD). These data were compared with the independent-samples *t* test. Skewed data are presented as median (IQR). They were analyzed with the Mann-Whitney *U* test. We summarized categorical variables as frequencies and percentages. Group differences were examined with the chi-square test or Fisher exact test, as appropriate. When Fisher exact test was used, chi-square statistics were not reported.

Associations between candidate predictors and LNM were examined using univariable and multivariable binary LR models, with effect estimates reported as ORs and 95% CIs.

Statistical analyses and missing-data handling were performed using R version 4.5.1 (R Core Team). Machine learning model development, performance evaluation, and model interpretation were performed using Python (Python Software Foundation) version 3.12.3 with scikit-learn (scikit-learn developers) version 1.4.2, XGboost (Distributed Machine Learning Community) version 2.1.1, lightGBM (Microsoft Corporation) version 4.5.0, and SHAP (Scott Lundberg) version 0.45.1.

### Ethical Considerations

This study was approved by the Ethics Committee of the First Affiliated Hospital of Harbin Medical University (approval No. 202452) and the Ethics Committee of Cancer Hospital, Chinese Academy of Medical Sciences, Shanxi Hospital (Shanxi Province Cancer Hospital; approval No. KY2024163). At the First Affiliated Hospital of Harbin Medical University, the requirement for informed consent was waived because of the retrospective study design. All patient-level data used for the present analysis were deidentified before analysis.

## Results

### Study Population and Data Characteristics

#### Patient Inclusion and Cohort Allocation

During the study period, 3642 patients with newly diagnosed CRC were screened for eligibility. Of these, 211 patients were excluded because they had received preoperative anticancer treatment, 567 because postoperative pathological LNM status could not be ascertained, and 139 because of synchronous or metachronous malignancies other than CRC. The final study cohort therefore comprised 2725 patients from 2 independent medical centers (Figure 1).

The internal development cohort included 1074 patients from the First Affiliated Hospital of Harbin Medical University. Stratified random splitting at a 7:3 ratio yielded 753 patients for model training and 321 for internal testing. The independent external validation cohort comprised 1651 patients from Shanxi Province Cancer Hospital.

**Figure 1.**
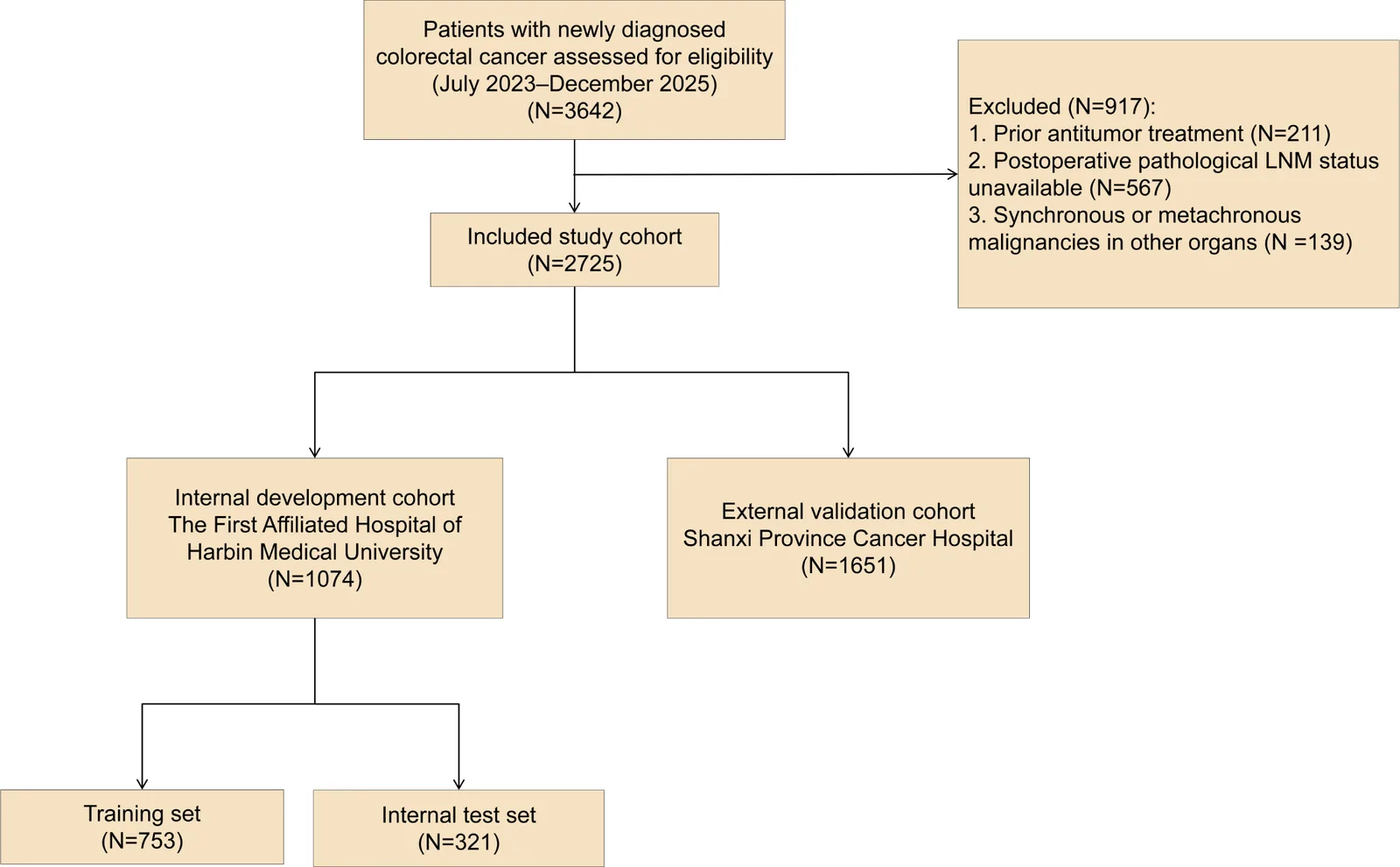
Study flowchart for patient screening, cohort allocation, model development, and validation. LNM: lymph node metastasis.

#### Baseline Characteristics and Missing Data

The development and external validation cohorts differed significantly in pCA19-9 (*P*=.004), SII (*P*=.03), residence (*P*<.001), family history (*P*<.001), smoking status (*P*=.03), and the number of lymph nodes examined (*P*=.01). No significant differences were observed in the other baseline characteristics, including LNM status (Table 1). No significant differences were observed between the training and internal test sets (Table S1 in Multimedia Appendix 1). Missingness was low overall, with missing proportions below 5% for all prespecified candidate predictors across the 3 data sets. The maximum missingness was 35/753 (4.65%) in the training set, 14/321 (4.36%) in the internal test set, and 78/1651 (4.72%) in the external validation set (Table S2 in Multimedia Appendix 1).

**Table 1.** Baseline characteristics of the development and external validation cohorts.

Characteristic^a^	Development cohort (n=1074)	External validation cohort (n=1651)	Statistics^b^	*P* value
Age, median (IQR) years	66.00 (60.00‐73.00)	67.00 (58.00‐73.00)	0.100^c^	.92
Sex, n (%)			2.880 (1)^d^	.09
Male	679 (63.2)	989 (59.9)		
Female	395 (36.8)	662 (40.1)		
Ethnicity, n (%)			3.650 (1)^d^	.06
Han	1059 (98.6)	1641 (99.4)		
Other	15 (1.4)	10 (0.6)		
Residence, n (%)			108.589 (1)^d^	<.001
Urban	768 (71.5)	848 (51.4)		
Rural	306 (28.5)	803 (48.6)		
BMI, kg/m², median (IQR)	23.88 (21.26‐26.22)	23.71 (21.57‐26.25)	-0.216^c^	.83
Smoking status, n (%)			6.943 (2)^d^	.03
Never smoking	874 (81.4)	1335 (80.9)		
Current smoking	187 (17.4)	272 (16.5)		
Former smoking	13 (1.2)	44 (2.7)		
Drinking status, n (%)			4.415^d^ (2)	.11
Never drinking	926 (86.2)	1392 (84.3)		
Current drinking	136 (12.7)	248 (15.0)		
Former drinking	12 (1.1)	11 (0.7)		
Family history, n (%)			115.977^d^ (1)	<.001
Yes	6 (0.6)	191 (11.6)		
No	1068 (99.4)	1460 (88.4)		
Comorbidities, n (%)			2.196^d^ (1)	.14
Yes	942 (87.7)	1414 (85.6)		
No	132 (12.3)	237 (14.4)		
Bowel obstruction, n (%)			3.417^d^ (1)	.07
Yes	215 (20.0)	283 (17.1)		
No	859 (80.0)	1368 (82.9)		
pCEA^e^, median (IQR) ng/mL	4.21 (2.35‐10.31)	4.47 (2.04‐9.59)	0.938^c^	.35
pCA19-9^f^, median (IQR) U/mL	9.96 (5.14‐19.32)	11.59 (6.24‐21.62)	-2.909^c^	.004
Neutrophil percentage, %	63.60 (56.02‐70.30)	62.40 (55.50‐69.85)	1.594^c^	.11
SII^g^, median (IQR)	598.63 (400.50‐993.44)	565.20 (392.04‐897.17)	2.163^c^	.03
AGR^h^, median (IQR)	1.46 (1.27‐1.66)	1.45 (1.29‐1.62)	0.857^c^	.39
Primary tumor site, n (%)			4.280 (2)^d^	.12
Rectum	454 (42.3)	670 (40.6)		
Left-sided colon	332 (30.9)	478 (29.0)		
Right-sided colon	288 (26.8)	503 (30.5)		
Maximum tumor diameter, median (IQR) cm	3.80 (3.00‐4.50)	3.50 (3.00‐5.00)	-1.895^c^	.06
cT^i^, n (%)			2.490 ^d^(3)	.48
cT1	65 (6.1)	82 (5.0)		
cT2	223 (20.8)	330 (20.0)		
cT3	713 (66.4)	1136 (68.8)		
cT4	73 (6.8)	103 (6.2)		
Gross tumor type, n (%)			3.191 (1)^d^	.07
Ulcerative or infiltrative	725 (67.5)	1169 (70.8)		
Protruding	349 (32.5)	482 (29.2)		
Histological type, n (%)			3.557^d^ (1)	.06
Adenocarcinoma	898 (83.6)	1425 (86.3)		
Mucinous adenocarcinoma or signet-ring cell carcinoma	176 (16.4)	226 (13.7)		
Tumor differentiation, n (%)			4.291^d^ (2)	.12
G1	51 (4.7)	58 (3.5)		
G2	830 (77.3)	1259 (76.3)		
G3 or G4	193 (18.0)	334 (20.2)		
Lymph node metastasis, n (%)			0.082 (1)^d^	.77
Yes	396 (36.9)	619 (37.5)		
No	678 (63.1)	1032 (62.5)		
Number of lymph nodes examined, median (IQR)	14.00 (12.00‐17.00)	14.00 (12.00‐17.00)	2.527^c^	.01

a Values are median (IQR) or n (%) unless otherwise indicated. The Mann-Whitney U test was used for continuous variables, and the chi-square test or Fisher exact test was used for categorical variables, as appropriate.

b Values are median (IQR) or n (%) unless otherwise indicated. The Mann-Whitney *U* test was used for continuous variables, and the chi-square test was used for categorical variables. Z denotes the standardized test statistic from the Mann-Whitney *U* test. Z values are reported for continuous variables, whereas chi-square values are reported for categorical variables, with degrees of freedom given in parentheses.

c Z value.

d *χ*² (df).

e pCEA: preoperative carcinoembryonic antigen.

f pCA19-9: preoperative carbohydrate antigen 19‐9.

g SII: systemic immune-inflammation index.

h AGR: albumin-to-globulin ratio.

i cT: clinical Tumor stage.

### Biopsy-Resection Agreement

Agreement between preoperative biopsy and postoperative resection pathology was moderate for both histological type and tumor differentiation. For histological type, the exact agreement rate was 88.5% (2392/2703), with an unweighted Cohen κ of 0.616 (95% CI 0.577‐0.653); 42.5% (248/583) of postoperative special histological types had been classified as adenocarcinoma on preoperative biopsy. For tumor differentiation, the exact agreement rate was 77% (2074/2692), with a linearly weighted Cohen κ of 0.521 (95% CI 0.488‐0.552); undergrading and overgrading occurred in 13.9% (374/2692) and 9.1% (244/3692) of cases, respectively (Table S3 in Multimedia Appendix 1).

### Predictor Selection

#### Univariable LR Analysis

Univariable analysis identified BMI, pCEA, bowel obstruction, maximum tumor diameter, gross tumor type, primary tumor site, clinical T stage, histological type, and tumor differentiation as significantly associated with LNM (Table S4 in Multimedia Appendix 1). Higher BMI and pCEA levels, bowel obstruction, right-sided colon or rectal location, mucinous adenocarcinoma or signet-ring cell carcinoma, and poorer tumor differentiation were associated with higher odds of LNM, whereas protruding gross tumor type was associated with lower odds. Compared with cT1, cT3 and cT4 were associated with substantially higher odds of LNM (OR 8.238 and 11.520, respectively; both *P*<.001). cT2 showed a lower odds estimate than cT1 in the univariable analysis (OR 0.186, 95% CI 0.043‐0.810; *P*=.03). The remaining candidate predictors were not significantly associated with LNM.

#### Multivariable LR Analysis

Multivariable LR identified BMI, pCEA, primary tumor site, cT, histological type, and tumor differentiation as factors independently associated with LNM (Table 2). Higher BMI (OR 1.154, 95% CI 1.095‐1.216; *P*<.001) and pCEA (OR 1.005, 95% CI 1.001‐1.010; *P*=.03) were associated with higher odds of LNM. Compared with left-sided colon tumors, right-sided colon tumors (OR 1.756, 95% CI 1.101‐2.801; *P*=.02) and rectal tumors (OR 1.838, 95% CI 1.196‐2.824; *P*=.005) also showed higher odds of LNM.

Clinical T stage showed the strongest associations: compared with cT1, cT3 and cT4 were associated with markedly higher odds of LNM (OR 11.798 and 17.743, respectively; both *P*<.001), whereas cT2 was not statistically significant (*P*=.10). Mucinous adenocarcinoma or signet-ring cell carcinoma was associated with higher odds than adenocarcinoma (OR 1.880, 95% CI 1.172‐3.017; *P*=.009). Compared with G1 differentiation, G2 (OR 5.122, 95% CI 1.082‐24.237; *P*=.04) and G3 or G4 (OR 13.593, 95% CI 2.755‐67.070; *P*=.001) were associated with higher odds of LNM. Gross tumor type, bowel obstruction, and maximum tumor diameter were no longer statistically significant after multivariable adjustment.

**Table 2.** Multivariable logistic regression analysis of factors associated with lymph node metastasis in the training set. Reference categories are shown directly in the table.

Variable	β coefficient	SE	Adjusted OR^a^ (95% CI)	*P* values
BMI, kg/m²	.143	0.027	1.154 (1.095‐1.216)	<.001
pCEA^b^, ng/mL	.005	0.002	1.005 (1.001‐1.010)	.03
Gross tumor type				
Ulcerative or infiltrative (Reference)				
Protruding	−.134	0.216	0.875 (0.573‐1.336)	.57
Bowel obstruction				
No (Reference)				
Yes	−.003	0.221	0.997 (0.647‐1.538)	.99
Maximum tumor diameter, cm	−.112	0.059	0.894 (0.797‐1.003)	.06
Primary tumor site				
Left-sided colon (Reference)				
Right-sided colon	.563	0.238	1.756 (1.101‐2.801)	.02
Rectum	.609	0.219	1.838 (1.196‐2.824)	.005
Clinical T stage				
cT1 (Reference)				
cT2	−1.335	0.811	0.263 (0.054‐1.291)	.100
cT3	2.468	0.581	11.798 (3.775‐36.866)	<.001
cT4	2.876	0.650	17.743 (4.963‐63.425)	<.001
Histological type				
Adenocarcinoma (Reference)				
Mucinous adenocarcinoma or signet-ring cell carcinoma	.631	0.241	1.880 (1.172‐3.017)	.009
Tumor differentiation				
G1 (Reference)				
G2	1.634	0.793	5.122 (1.082‐24.237)	.04
G3 or G4	2.610	0.814	13.593 (2.755‐67.070)	.001

a OR: odds ratio.

b pCEA: preoperative carcinoembryonic antigen.

### Model Performance and Model Selection

#### Discrimination and Classification Performance

The discrimination and classification performance of the 7 candidate models is summarized in Table 3 and Figure 2. In the internal test set, AUROCs ranged from 0.708 to 0.809. The RF model achieved an AUROC of 0.803 (95% CI 0.756‐0.849), with an accuracy of 0.720, sensitivity of 0.763, specificity of 0.695, and *F*_1_ score of 0.667. In the external validation set, RF showed the highest AUROC among the candidate models (0.776, 95% CI 0.755‐0.797), with an accuracy of 0.687, sensitivity of 0.677, specificity of 0.693, and *F*_1_ score of 0.618.

Pairwise DeLong comparisons showed that, in the internal test set, the RF AUROC differed significantly only from that of the DT after Holm adjustment. In the external validation set, RF showed significantly higher AUROCs than SVM, LR, DT, and NB, whereas the differences versus XGBoost and LightGBM were not statistically significant after adjustment (Table S6 in Multimedia Appendix 1). Using the RF training-derived Youden operating point of 0.545, the PPV and NPV were 0.592 and 0.834 in the internal test set and 0.569 and 0.781 in the external validation set, respectively. The corresponding false-positive and false-negative counts were 62 and 28 in the internal test set and 317 and 200 in the external validation set. Threshold-dependent classification metrics are summarized in Table 3, while PPV, NPV, and full confusion-matrix counts are provided in Table S7 in Multimedia Appendix 1.

**Table 3.** Predictive performance of the candidate machine learning models in the training, internal test, and external validation sets.

Model	Data set^a^	AUROC^b^ (95% CI)	Accuracy (95% CI)^c^	Sensitivity (95% CI)^c^	Specificity (95% CI)^c^	*F*_1_ score (95% CI)^c^
RF^d^	Training	0.862 (0.837‐0.887)	0.768 (0.735‐0.799)	0.809 (0.760‐0.853)	0.743 (0.703‐0.785)	0.720 (0.680‐0.762)
RF	Internal test	0.803 (0.756‐0.849)	0.720 (0.670‐0.769)	0.763 (0.677‐0.834)	0.695 (0.630‐0.759)	0.667 (0.593‐0.731)
RF	External validation	0.776 (0.755‐0.797)	0.687 (0.664‐0.709)	0.677 (0.644‐0.716)	0.693 (0.665‐0.719)	0.618 (0.588‐0.649)
SVM^e^	Training	0.798 (0.767‐0.832)	0.709 (0.679‐0.741)	0.827 (0.779‐0.876)	0.640 (0.597‐0.684)	0.677 (0.635‐0.717)
SVM	Internal test	0.790 (0.744‐0.836)	0.704 (0.654‐0.756)	0.847 (0.776‐0.914)	0.621 (0.559‐0.693)	0.678 (0.612‐0.735)
SVM	External validation	0.751 (0.725‐0.775)	0.652 (0.629‐0.676)	0.732 (0.696‐0.768)	0.605 (0.576‐0.634)	0.612 (0.584‐0.641)
LR^f^	Training	0.754 (0.717‐0.789)	0.664 (0.633‐0.699)	0.827 (0.783‐0.873)	0.568 (0.526‐0.611)	0.645 (0.606‐0.683)
LR	Internal test	0.778 (0.724‐0.828)	0.713 (0.660‐0.766)	0.856 (0.794‐0.918)	0.631 (0.566‐0.702)	0.687 (0.625‐0.748)
LR	External validation	0.671 (0.644‐0.696)	0.593 (0.571‐0.616)	0.703 (0.670‐0.737)	0.527 (0.495‐0.557)	0.564 (0.538‐0.597)
DT^g^	Training	0.942 (0.927‐0.956)	0.849 (0.821‐0.874)	0.863 (0.821‐0.903)	0.840 (0.808‐0.876)	0.808 (0.771‐0.843)
DT	Internal test	0.708 (0.649‐0.766)	0.651 (0.598‐0.704)	0.644 (0.552‐0.738)	0.655 (0.593‐0.727)	0.576 (0.493‐0.652)
DT	External validation	0.675 (0.646‐0.699)	0.637 (0.612‐0.661)	0.572 (0.529‐0.610)	0.675 (0.648‐0.706)	0.541 (0.505‐0.573)
XGBoost^h^	Training	0.871 (0.846‐0.896)	0.786 (0.758‐0.817)	0.809 (0.766‐0.855)	0.773 (0.736‐0.810)	0.736 (0.696‐0.779)
XGBoost	Internal test	0.790 (0.741‐0.833)	0.723 (0.670‐0.769)	0.729 (0.644‐0.802)	0.719 (0.655‐0.773)	0.659 (0.590‐0.719)
XGBoost	External validation	0.768 (0.747‐0.789)	0.681 (0.660‐0.704)	0.653 (0.617‐0.688)	0.698 (0.670‐0.726)	0.605 (0.575‐0.636)
NB^i^	Training	0.798 (0.767‐0.829)	0.707 (0.672‐0.740)	0.856 (0.813‐0.896)	0.619 (0.575‐0.665)	0.683 (0.640‐0.718)
NB	Internal test	0.809 (0.766‐0.854)	0.732 (0.682‐0.777)	0.890 (0.828‐0.943)	0.640 (0.571‐0.706)	0.709 (0.654‐0.767)
NB	External validation	0.734 (0.708‐0.758)	0.643 (0.621‐0.667)	0.743 (0.710‐0.774)	0.583 (0.552‐0.613)	0.610 (0.580‐0.637)
LightGBM^j^	Training	0.872 (0.849‐0.895)	0.780 (0.752‐0.807)	0.831 (0.788‐0.871)	0.749 (0.711‐0.788)	0.736 (0.698‐0.772)
LightGBM	Internal test	0.791 (0.752‐0.835)	0.717 (0.673‐0.765)	0.746 (0.664‐0.822)	0.700 (0.639‐0.761)	0.659 (0.597‐0.726)
LightGBM	External validation	0.769 (0.751‐0.792)	0.680 (0.660‐0.701)	0.670 (0.640‐0.710)	0.685 (0.658‐0.714)	0.611 (0.583‐0.643)

a Data set sizes were 753 for training, 321 for internal testing, and 1651 for external validation.

b AUROC: area under the receiver operating characteristic curve.

c Classification metrics were calculated using model-specific training-derived operating points selected by maximizing the Youden index in the training set: RF, 0.545; SVM, 0.316; LR, 0.343; DT, 0.429; XGBoost, 0.439; NB, 0.208; and LightGBM, 0.413. These operating points were applied unchanged to the internal test and external validation sets and were used for performance evaluation rather than as validated clinical decision cutoffs. Values are presented to 3 decimal places; full-precision values were used in the analyses. PPV, NPV, and full confusion-matrix counts are reported in Table S7 in Multimedia Appendix 1.

d RF: random forest.

e SVM: support vector machine.

f LR: logistic regression.

g DT: decision tree.

h XGBoost: extreme gradient boosting.

i NB: Naive Bayes.

j LightGBM: light gradient boosting machine.

**Figure 2.**
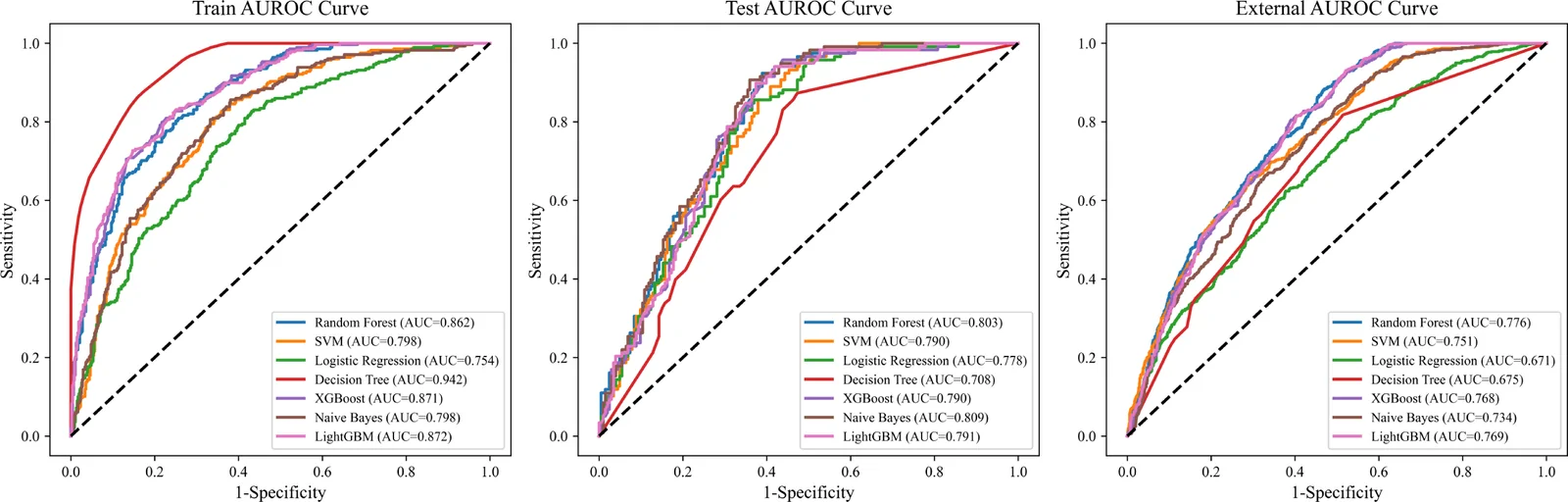
Area under receiver operating characteristic curves of the 7 candidate machine learning models in the training, internal test, and external validation sets. AUROC: area under receiver operating characteristic; LightGBM: light gradient boosting machine; SVM: support vector machine; XGBoost: extreme gradient boosting.

#### Calibration and Clinical Utility

Calibration curves are shown in Figure 3, and quantitative calibration statistics, including the Brier score, calibration intercept, calibration slope, and observed-to-expected ratio, are reported in Multimedia Appendix 1, Table S8 in Multimedia Appendix 1. Corresponding calibration results from the sensitivity analyses are provided in Table S10 in Multimedia Appendix 1. For the RF model, the Brier score was 0.179 in the internal test set and 0.190 in the external validation set. The calibration intercepts were −0.176 and −0.120, the calibration slopes were 1.161 and 1.061, and the observed-to-expected ratios were 0.806 and 0.803, respectively. These estimates indicated reasonably stable calibration across the 2 validation sets, although the CIs should be considered when interpreting precision.

DCA is shown in Figure 4, and the corresponding model-specific net-benefit threshold-probability ranges are summarized in Table S9 in Multimedia Appendix 1. The lower bound of 0.05 was the prespecified lower limit of the display interval; these ranges were exploratory and should not be interpreted as validated clinical decision thresholds.

**Figure 3.**
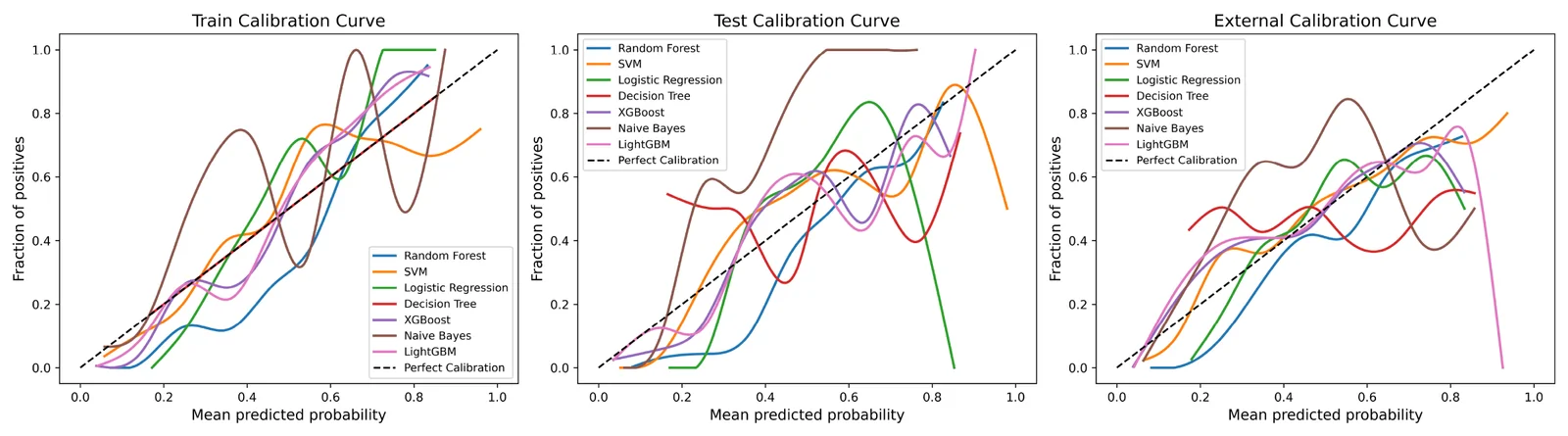
Calibration curves of the 7 candidate machine learning models in the training, internal test, and external validation sets. AUROC: area under receiver operating characteristic; LightGBM: light gradient boosting machine; SVM: support vector machine; XGBoost: extreme gradient boosting.

**Figure 4.**
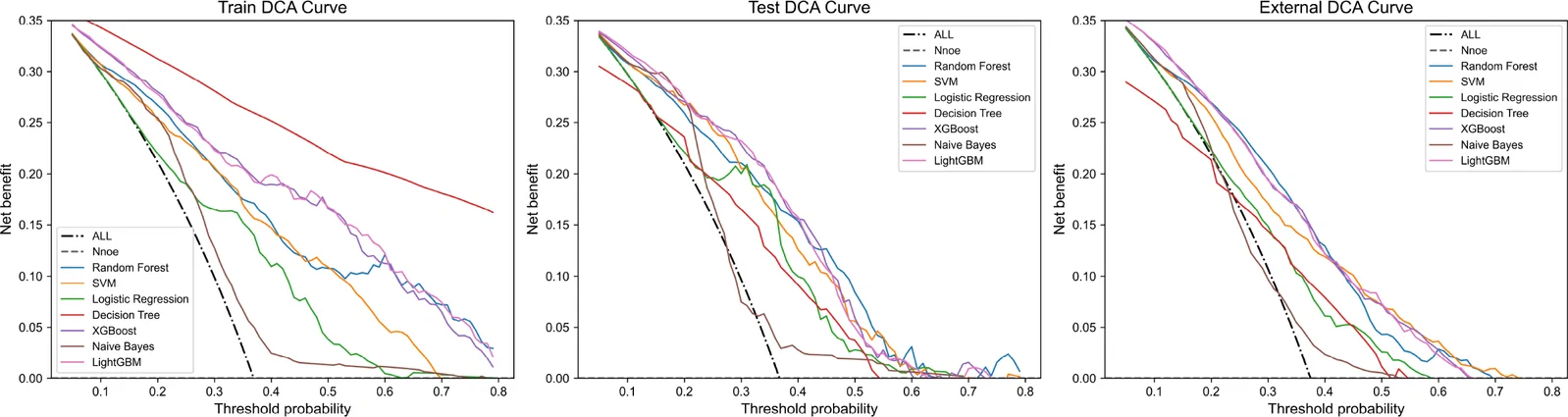
Decision curve analysis of the 7 candidate machine learning models in the training, internal test, and external validation sets. AUROC: area under the receiver operating curve; DCA: decision curve analysis; LightGBM: light gradient boosting machine; XGBoost: extreme gradient boosting.

#### Final Model Selection

Considering discrimination, classification performance, calibration, and clinical utility in the internal test set, RF was selected as the final model before external validation. RF achieved the highest AUROC in the external validation set (0.776), while maintaining relatively balanced sensitivity (0.677) and specificity (0.693). Its calibration slope was close to 1 in both the internal test (1.161) and external validation (1.061) sets. Although the external-validation AUROC differences between RF and XGBoost or LightGBM were not statistically significant after Holm adjustment, RF showed a favorable overall balance across the prespecified internal-test criteria and was therefore selected for subsequent external validation and interpretability analyses.

### Model Interpretation

#### Global Feature Importance

SHAP analysis showed that clinical T stage had the greatest overall contribution to the RF model, followed by BMI, tumor differentiation, pCEA, the left-sided colon indicator, histological type, and the rectum indicator (Figure S1 in Multimedia Appendix 1). The SHAP summary plot further showed that higher clinical T stage, BMI, poorer tumor differentiation, higher pCEA, and special histological type generally shifted model predictions toward LNM positivity (Figure 5). In contrast, the left-sided colon indicator was predominantly associated with negative SHAP values relative to the right-sided colon reference category.

**Figure 5.**
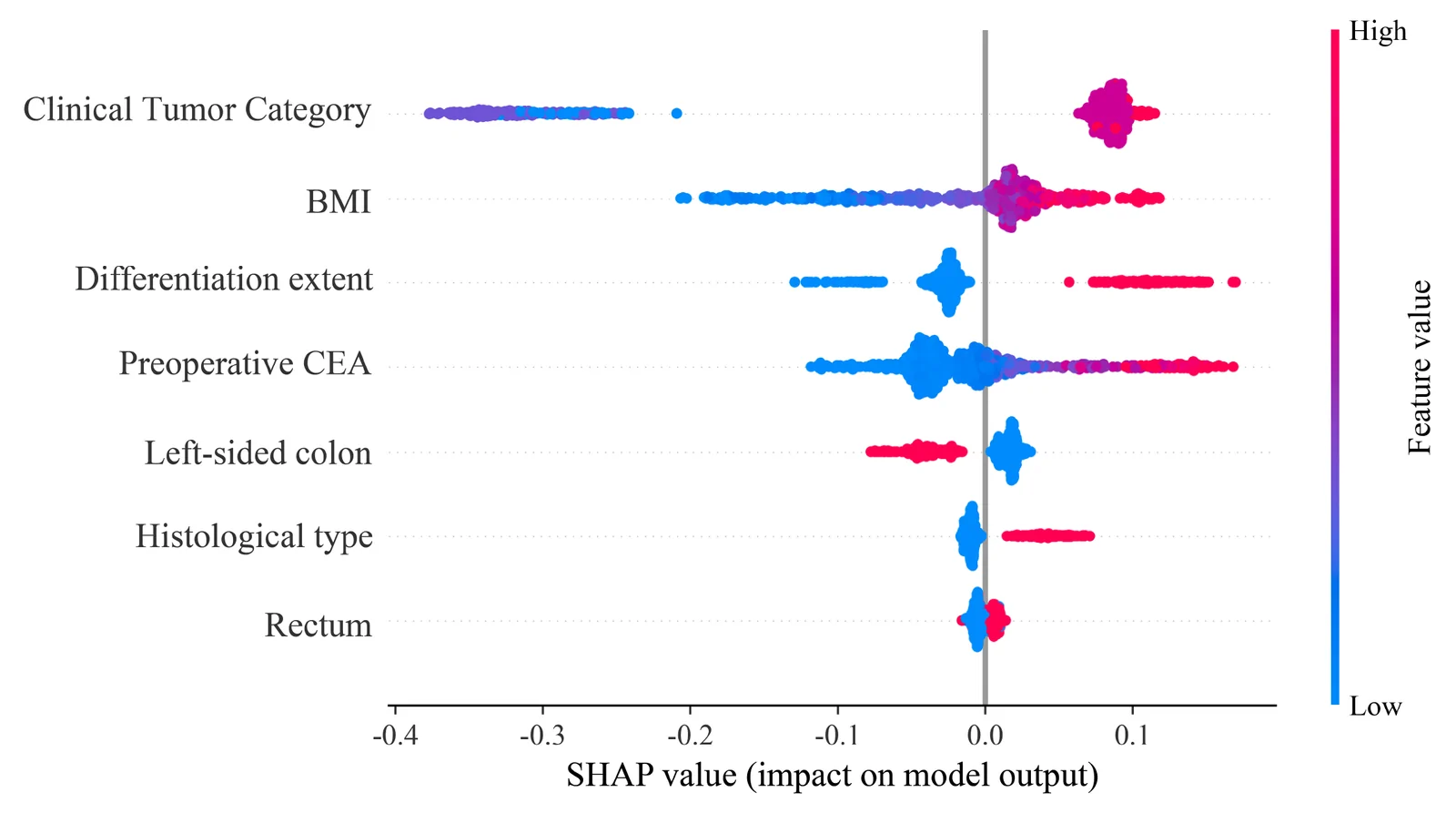
Shapely additive explanations summary plot of the encoded features in the final random forest model. CEA: carcinoembryonic antigen; SHAP: Shapely additive explanations.

#### Individual Prediction and Feature Dependence

For the representative patient shown in Figure S2 in Multimedia Appendix 1, the RF model estimated an LNM probability of 0.804, compared with a baseline probability of 0.504. G3 or G4 differentiation, cT3 stage, elevated pCEA (22.9 ng/mL), and higher BMI (25.9 kg/m²) contributed positively to the prediction, with tumor differentiation showing the largest individual contribution.

SHAP dependence plots further demonstrated distinct patterns across predictors (Figure S3 in Multimedia Appendix 1). Higher BMI and pCEA were generally associated with increasingly positive SHAP values. cT1 and cT2 contributed predominantly toward LNM-negative predictions, whereas cT3 and cT4 contributed toward LNM-positive predictions. G3 or G4 differentiation and mucinous adenocarcinoma or signet-ring cell carcinoma also showed predominantly positive contributions. For tumor location, the left-sided colon indicator was associated with lower SHAP values when present, whereas the rectum indicator showed comparatively smaller and more variable contributions.

### Sensitivity Analyses

Sensitivity analyses showed that the RF model based on the 6 selected predictors performed similarly to the model incorporating all prespecified candidate predictors. The AUROC differences were not statistically significant in either the internal test set (0.803 vs 0.833; *P*=.15) or the external validation set (0.776 vs 0.787; *P*=.22). The all-candidate-predictor model showed slightly higher accuracy and lower Brier scores, but the overall performance profiles of the 2 models were comparable.

In contrast, the 6-predictor RF model showed significantly better discrimination than the model using clinical T stage alone, with AUROC differences of 0.091 in the internal test set and 0.069 in the external validation set (both *P*<.001). Calibration and decision curve analyses were consistent with improved probabilistic accuracy and broader exploratory net-benefit ranges for the multivariable model than for clinical T stage alone (Table S10 and Figure S4 in Multimedia Appendix 1).

## Discussion

### Principal Findings

In this multicenter retrospective study, we developed and externally validated a machine learning model for preoperative estimation of LNM risk in patients with CRC using routinely available clinical information. Six preoperative predictors—BMI, preoperative carcinoembryonic antigen (CEA), primary tumor site, clinical T stage, histological type, and tumor differentiation—were retained through the prespecified predictor-selection procedure. Among the 7 candidate algorithms, RF was selected as the final model based on its overall performance in the internal test set, with particular consideration of discrimination and the balance of threshold-dependent classification performance. The model achieved an AUROC of 0.803 in the internal test set and maintained an AUROC of 0.776 in the independent external validation cohort. In external validation, sensitivity and specificity were 0.677 and 0.693, respectively, while the calibration slope was 1.061 and the Brier score was 0.190, indicating that the model retained moderate discrimination with relatively stable classification and calibration performance when transported to an independent center.

An important finding was that the predictive information captured by the final model extended beyond clinical T stage alone. Although clinical T stage was the most influential predictor in the SHAP analysis, the clinical T stage–only RF model showed substantially lower discrimination than the final 6-predictor model in both the internal test set (AUROC 0.712 vs 0.803) and the external validation set (AUROC 0.707 vs 0.776). The corresponding AUROC differences of 0.091 and 0.069 were statistically significant in both datasets (both *P*<.001). These findings indicate that clinical T stage provides an important foundation for LNM risk estimation, but that BMI, preoperative CEA, tumor location, histological type, and tumor differentiation contribute additional complementary information for distinguishing individual risk.

Conversely, expanding the RF model from the selected 6 predictors to all prespecified candidate predictors did not result in a statistically significant improvement in discrimination. The all-predictor model yielded AUROCs of 0.833 and 0.787 in the internal test and external validation sets, respectively, compared with 0.803 and 0.776 for the 6-predictor model; the corresponding differences were not statistically significant (*P*=.15 and *P*=.22, respectively). Although the expanded model showed small numerical improvements in some performance measures, the absence of a clear gain in discrimination suggests that substantially increasing the number of predictors added limited incremental predictive information. The selected 6-predictor model therefore represents a relatively parsimonious approach that preserves most of the predictive information available from the broader set of routinely collected variables.

The SHAP analysis showed that the final RF model used information from several predictor domains. cT contributed most strongly, followed by BMI, tumor differentiation, pCEA, primary tumor site, and histological type. Higher cT, poorer tumor differentiation, higher BMI, and elevated pCEA generally shifted predictions toward a higher probability of LNM. These patterns describe model associations and should not be interpreted as causal effects or as evidence that the model explicitly identifies causal interactions.

### Interpretation of Key Predictors

This finding is consistent with previous studies identifying deeper tumor invasion as a strong correlate of LNM in CRC [2,8,9]. The strong contribution of cT in the present model is therefore consistent with its established relationship to local tumor invasion and regional nodal spread.

Tumor differentiation was also an important predictor in the model. In our study, differentiation was assessed on preoperative endoscopic biopsy. Poor differentiation has been associated with an increased risk of LNM, particularly in early CRC [10]. However, limited tissue sampling may prevent preoperative biopsy from fully representing the tumor’s overall differentiation. Barresi et al [11] reported concordant grading in approximately 75% of CRCs using a grading system based on poorly differentiated clusters, and the biopsy-derived grade was associated with nodal involvement and pTNM stage. Although their grading system differed from that used in our study, these findings support the clinical relevance of adverse histological features identified on preoperative biopsy. In our biopsy-resection analysis, tumor differentiation showed 77.0% (2074/2692) exact agreement, with a linearly weighted Cohen κ of 0.521, and 13.9% (374/2692) of tumors were undergraded on preoperative biopsy. Biopsy-reported differentiation therefore provides useful preoperative information, although it may not fully capture the highest-grade component of the tumor. Poor differentiation on biopsy may serve as a meaningful high-risk feature, whereas a more favorable grade should be interpreted with awareness of sampling limitations.

Histological type showed a similar pattern. Mucinous adenocarcinoma and signet-ring cell carcinoma have distinct clinicopathological characteristics and are generally associated with more aggressive disease than conventional adenocarcinoma [12]. In our cohort, histological type showed 88.5% (2392/2703) exact agreement between preoperative biopsy and resection pathology, with a Cohen κ of 0.616. However, 42.5% (248/583) of tumors ultimately classified as mucinous adenocarcinoma or signet-ring cell carcinoma on the resection specimen had not been identified as a special histological type on preoperative biopsy. Previous studies have also reported that mucinous colorectal adenocarcinoma may be missed on initial endoscopic biopsy, reflecting the limitations of tissue sampling [13]. Importantly, despite incomplete biopsy-resection agreement, biopsy-derived histological type remained independently associated with LNM and contributed to the RF predictions, supporting its value as a routinely available preoperative predictor.

pCEA was independently associated with LNM, consistent with previous studies identifying elevated CEA as a risk factor for nodal involvement in CRC [14]. Our findings support pCEA as a routinely available preoperative marker for LNM risk prediction.

We found that right-sided colon and rectal tumors were associated with higher odds of LNM than left-sided colon tumors. CRCs arising at different anatomical sites show distinct clinicopathological and biological characteristics [15,16], which may partly explain the observed differences in nodal involvement. Our findings suggest that primary tumor site provides additional information for preoperative LNM risk prediction.

BMI was independently associated with LNM and ranked highly in the SHAP analysis. Obesity-related phenotypes have been linked to CRC development and progression [17,18], although BMI is only a simple proxy for overall adiposity. The observed association should not be interpreted as causal; BMI may instead function as a readily available patient-level marker correlated with other biological or clinical factors.

Taken together, these routinely available preoperative features capture complementary information on tumor extent, morphology, circulating markers, anatomical site, and patient characteristics, providing a broader basis for individualized LNM risk estimation.

### Comparison With Previous Studies

Conventional imaging remains the foundation of preoperative staging in CRC, with CT commonly used for colon cancer and pelvic MRI for rectal cancer. Imaging provides important information on local tumor extent, as also reflected by the strong contribution of cT in our model. However, direct assessment of nodal metastasis still relies largely on lymph node size and morphological characteristics, which have limited accuracy for distinguishing metastatic from benign nodes. A meta-analysis of MRI-based nodal staging in rectal cancer reported only moderate diagnostic performance across different size and morphological criteria [19], while a recent systematic review similarly found limited performance for preoperative N staging in colon cancer using MRI or 18F-fluorodeoxyglucose positron emission tomography/computed tomography (18F-FDG PET/CT) [20]. These limitations provide a rationale for complementing conventional imaging with additional preoperative clinical and pathological information when estimating patient-level LNM risk.

Previous CRC metastasis prediction models have commonly included cancer stage, tumor differentiation, histological type, CEA, and demographic factors [21]. However, external validation has been limited: only 4 of 12 models identified in an umbrella review had undergone external validation, and calibration was incompletely reported [21]. More recent meta-analytic evidence also found higher reported AUROCs in studies without external validation [5]. Our model used routinely available preoperative variables and was evaluated in a large independent external cohort.

Quantitative imaging approaches, including radiomics and deep learning, aim to extract information beyond conventional visual assessment. A recent systematic review and meta-analysis reported promising performance of radiomics for preoperative LNM prediction in CRC, although substantial heterogeneity across studies remains [22]. Multimodal models further combine imaging-derived features with clinical variables to capture complementary information. However, the added value of these features may not always persist in external validation. In a multicenter rectal cancer study, Li et al [23] found that a clinical-radiomics model outperformed the clinical model in the training and internal validation cohorts, but this advantage was not maintained in the external cohort.

Taken together, previous studies illustrate a trade-off between the amount of predictive information incorporated and the complexity of data acquisition and model implementation. Differences in study populations, predictor sets, imaging modalities, and validation strategies make direct comparison of reported AUROCs across studies difficult [5,21,22]. In this context, our study focused on routinely available preoperative information, including clinical staging, laboratory markers, and biopsy-derived pathological features, without requiring radiomic feature extraction or additional molecular testing. The selected model was evaluated in a large independent external cohort, and sensitivity analyses showed that the 6-predictor model provided additional discriminatory information beyond cT alone, whereas expanding the model to all candidate predictors did not significantly improve discrimination. These findings support a relatively parsimonious model for preoperative LNM risk estimation, but they do not establish superiority over every existing clinical model.

### Clinical Implications and Intended Use

Preoperative nodal assessment remains imperfect in CRC, leaving uncertainty even after conventional imaging and clinical staging. Our model addresses this uncertainty by integrating routinely available clinical, laboratory, imaging-derived, and biopsy-based information into an individualized estimate of pathological LNM risk. Individualized risk estimation is an important application of clinical prediction models because it translates multiple patient characteristics into a probability that can be interpreted at the patient level [24]. In our study, the 6-predictor model also provided additional discriminatory information beyond cT alone, suggesting that routinely collected features can complement conventional assessment of local tumor extent.

A practical advantage of the model is that all predictors are already available during routine preoperative assessment and do not require dedicated radiomic feature extraction, molecular assays, or additional specialized examinations. This may simplify future evaluation in routine-care settings, although the completeness and reliability of these inputs, as well as transportability across centers, must be confirmed before implementation [25,26].

The model was developed in patients with newly diagnosed CRC who proceeded directly to radical surgery without preoperative anticancer treatment, and its intended use is therefore preoperative LNM risk stratification in a similar direct-surgery population. Its performance has not been validated in patients being considered for endoscopic resection, local excision, neoadjuvant therapy, or nonoperative management. The predicted probability is not intended to function as an isolated treatment criterion. A possible future workflow would be to calculate the probability after routine preoperative data collection and initial imaging review, then use discordance between the model estimate and the clinical assessment to prompt verification of the input data, focused radiology or pathology review, or multidisciplinary discussion. This workflow is a proposed implementation scenario for prospective evaluation, not a validated decision rule.

### Strengths and Limitations

This study has several strengths. It included a large multicenter cohort with independent external validation, and all predictors were routinely available before surgery without requiring radiomics, molecular testing, or additional specialized examinations. Model evaluation included discrimination, calibration, DCA, threshold-dependent performance, and SHAP interpretation. Biopsy-resection agreement and sensitivity analyses against cT alone and the full predictor set provided additional evidence regarding predictor reliability and model robustness.

Several limitations should be acknowledged. First, the retrospective design and inclusion of only 2 centers in China may limit generalizability. Second, eligibility was restricted to patients with newly diagnosed CRC who underwent direct radical resection with regional lymph node dissection without preoperative anticancer treatment; therefore, the findings should not be extrapolated to patients undergoing neoadjuvant therapy, endoscopic resection, local excision, or nonoperative management. Patients with unavailable postoperative pathological LNM status were excluded, which may have introduced selection bias. Third, clinical regional lymph node category (cN category) was not included in the prespecified predictor set; therefore, the model was not directly benchmarked against clinical regional lymph node category-based assessment. The model also depends partly on imaging-derived cT, and its performance may vary with imaging quality, reader expertise, staging protocols, laboratory measurement, pathology processing, lymph-node yield, and biopsy practices across centers. Biopsy-derived differentiation and histological type showed imperfect agreement with resection pathology and remain subject to sampling limitations. Comorbidities were available only as a binary clinical-record variable without harmonized disease-specific definitions across centers. Early-stage tumors, particularly cT1-cT2 disease, were relatively underrepresented, and subgroup or fairness analyses were not performed. Finally, the training-derived evaluation operating point, DCA ranges, and threshold-dependent classifications were exploratory; no decision-impact or patient-outcome benefit was established. These factors define the current validation boundaries of the model: it has been evaluated in a direct-surgery population from 2 centers in China, with limited representation of early-stage disease, and its performance has not been established in other treatment pathways or broader clinical settings. Prospective multicenter validation across diverse populations and settings is required.

### Conclusions

We developed and externally validated a 6-predictor RF model for preoperative LNM risk estimation in patients with newly diagnosed CRC who proceed directly to radical resection with regional lymph node dissection without preoperative anticancer treatment. The model used routinely available clinical, laboratory, imaging-derived, and biopsy-based information and showed moderate discrimination in an independent external cohort, with predictive information beyond cT alone. Its intended role is adjunctive, research-stage risk stratification rather than replacement of imaging, pathology, or clinical judgment. Further prospective validation in broader populations, particularly patients with early-stage disease and those managed through different treatment pathways, is required before clinical implementation.

## Supplementary material

10.2196/101162 Multimedia Appendix 1 Supplementary tables and figures for model development, validation, interpretation, and sensitivity analyses.

10.2196/101162 Multimedia Appendix 2 Fitted random forest model, prediction script, README, and software requirements.

## Abbreviations

18F-FDG PET/CT: 18F-fluorodeoxyglucose positron emission tomography/computed tomography
AGR: albumin-to-globulin ratio
AUROC: area under the receiver operating characteristic curve
CEA: carcinoembryonic antigen
CRC: colorectal cancer
CT: computed tomography
cT: clinical tumor stage
cT1: clinical Tumor1 stage
cT2: clinical Tumor2 stage
cT3: clinical Tumor3 stage
cT4: clinical Tumor4 stage
DCA: decision curve analysis
DT: decision tree
GLOBOCAN: Global Cancer Observatory
LightGBM: light gradient-boosting machine
LNM: lymph node metastasis
LR: logistic regression
MRI: magnetic resonance imaging
NB: naive Bayes
NPV: negative predictive value
OR: odds ratio
pCA19-9: preoperative carbohydrate antigen 19‐9
pCEA: preoperative carcinoembryonic antigen
PPV: positive predictive value
RF: random forest
ROC: receiver operating characteristic
SHAP: Shapley additive explanations
SII: systemic immune-inflammation index
SVM: support vector machine
XGBoost: extreme gradient boosting
