# Supplementary material for: Preoperative Prediction of Lymph Node Metastasis in Colorectal Cancer Using Routinely Collected Clinical Data: Multicenter Retrospective Machine Learning Model Development and External Validation Study
Source: JMIR Cancer. 2026 Oct 7;12:e101162. doi: 10.2196/101162 (PMC13644950; doi:10.2196/101162)
Supplement: Supplementary file 1 [file cancer-v12-e101162-s002.pdf]

Multimedia Appendix 2. Supplementary materials for implementation of the random forest model.

README.md

```
# Random Forest Model for Preoperative Lymph Node Metastasis Probability Estimation
## Implementation Files
- `the fitted random forest model` — fitted random forest model used to generate the predicted probability of pathologically confirmed regional lymph node metastasis (LNM).
- `predict_lnm.py` — single-patient prediction script that validates the required inputs, applies the predefined encoding and feature order, loads the fitted model, and generates the model outputs. It does not retrain the model.
- `requirements.txt` — Python runtime dependencies for the released implementation.
- `README.md` — model input, execution, output, and interpretation instructions.
By default, `predict_lnm.py` loads `the fitted random forest model` from the directory containing the script. A different path to the same supplied model implementation may be specified with `--model`.
## Intended Population
The model is intended for patients with newly diagnosed colorectal cancer who proceed directly to radical resection with regional lymph node dissection without preoperative anticancer treatment.
The model has not been validated for patients being considered for endoscopic resection, local excision, neoadjuvant therapy, or nonoperative management.
## Required Clinical Inputs
Complete values for all 6 clinical predictors are required before prediction.
| Clinical predictor | Required input | Unit / accepted categories | Model encoding |
|---|---|---|---|
| Body mass index | `BMI`; CLI argument: `--bmi`; continuous numerical value | kg/m² | Retained as a continuous variable |
| Preoperative carcinoembryonic antigen | `pCEA`; CLI argument: `--pcea`; continuous numerical value | ng/mL | Retained as a continuous variable |
| Primary tumor site | CLI argument: `--site` | `right-sided colon`; `left-sided colon`; `rectum` | Nominal categorical predictor. Right-sided colon is the reference category. Left-sided colon and rectum are represented by separate dummy indicators. |
| Clinical T stage | CLI argument: `--ct` | `cT1`; `cT2`; `cT3`; `cT4` | `cT1=0`; `cT2=1`; `cT3=2`; `cT4=3` |
| Histological type, from preoperative endoscopic biopsy pathology | CLI argument: `--histology` | `adenocarcinoma`; `mucinous adenocarcinoma/signet-ring cell carcinoma` | `adenocarcinoma=0`; `mucinous adenocarcinoma/signet-ring cell carcinoma=1` |
| Tumor differentiation, from preoperative endoscopic biopsy pathology | CLI argument: `--differentiation` | `G1`; `G2`; `G3/G4` | `G1=0`; `G2=1`; `G3/G4=2` |
For primary tumor site, right-sided colon is encoded as both site indicators equal to 0, left-sided colon as the left-sided colon indicator equal to 1, and rectum as the rectum indicator equal to 1. Primary tumor site is a nominal categorical predictor. The dummy variables do not represent an ordinal risk gradient.
The script rejects nonfinite BMI or pCEA values, BMI values less than or equal to 0, pCEA values less than 0, and unrecognized categorical values.
## Relationship Between Clinical Inputs and Model Features
The model requires 6 clinical predictors. After one-hot encoding of primary tumor site, these 6 clinical inputs correspond to 7 encoded features supplied to the fitted random forest model.
The fitted model requires the following feature names in this fixed order:
1. `BMI`
2. `pCEA`
3. `Site_[redacted]`
4. `Site_[redacted]`
5. `cT Stage`
6. `Histology`
7. ` Differentiation extent`
```

The leading space in ` Differentiation extent` is part of the feature name stored in the fitted model and must be retained. The internal feature names are used only for compatibility with the fitted model.

Missing Values

The released implementation does not include the original training-time imputation object. The prediction script does not reproduce the training-time missing-data imputation procedure. Complete values for all 6 predictors are therefore required for application of the released model.

All 6 command-line arguments are required. The script does not automatically impute missing values and does not produce a prediction when the required inputs fail its validation checks.

Software Requirements

The model-development environment used Python 3.12.3. The supplied `requirements.txt` specifies:

```text
scikit-learn==1.4.2
pandas>=2.0,<3.0
numpy>=1.24,<2.0
joblib>=1.2,<2.0
```

Install the supplied requirements with:

```bash
python -m pip install -r requirements.txt
```

The fitted model was persisted with scikit-learn 1.4.2. Use the specified scikit-learn version because persisted scikit-learn models may not be compatible across versions.

Running a Prediction

Place `the fitted random forest model`, `predict_lnm.py`, and `requirements.txt` in the same directory, then run:

```bash
python predict_lnm.py --bmi 25.9 --pcea 22.9 --site "rectum" --ct "cT3" --histology "adenocarcinoma" --differentiation "G3/G4"
```

To provide the supplied model through an explicit path, add:

```bash
--model /path/to/the fitted random forest model
```

Prediction Output

The script prints a JSON object containing:

- `predicted_lnm_probability` — the predicted probability of pathologically confirmed regional LNM; this is the primary model output.
- `threshold_full_precision` — `0.5445649716955735`.
- `threshold_rounded_3dp` — `0.545`.
- `model_classification` — `High risk` when the predicted probability is greater than or equal to the full-precision operating point and `Low risk` otherwise.
- `note` — states that the threshold-based classification is research-stage and is not a validated clinical treatment threshold.

Intended Use and Interpretation

The model is intended for research-stage adjunctive patient-level LNM risk stratification within the direct-surgery population described above. The model output should not be used as a stand-alone criterion for treatment selection. It does not replace imaging assessment, pathological evaluation, or clinical judgment.

predict_lnm.py

```
#!/usr/bin/env python3
"""
Usable implementation of the final random-forest model for preoperative
prediction of pathological lymph node metastasis (LNM) in colorectal cancer.
This script:
1. accepts the 6 preoperative predictors used in the final model;
2. applies the exact variable encoding used for model development;
3. loads the fitted random-forest model;
4. returns the predicted probability of LNM;
5. applies the training-derived Youden threshold.
Intended population:
- This implementation is intended for patients with newly diagnosed colorectal
cancer who proceed directly to radical resection with regional lymph node
dissection without preoperative anticancer treatment.
- It has not been validated for patients being considered for endoscopic
resection, local excision, neoadjuvant therapy, or nonoperative management.
Important:
- This implementation is for research use and external validation within the
intended population described above.
- The classification threshold is not a validated clinical treatment threshold.
- The supplied fitted model does not contain the training-time imputation object.
Therefore, all 6 predictor values must be available for prediction.
"""
from __future__ import annotations
import argparse
import json
from pathlib import Path
from typing import Union
import joblib
import numpy as np
import pandas as pd
MODEL_FILENAME = "RF_LNM_model.pkl"
# Full-precision training-derived RF Youden threshold.
# Reported in the manuscript as 0.545 after rounding to 3 decimal places.
RF_THRESHOLD = 0.5445649716955735
# Feature names must exactly match the names stored in the fitted model.
MODEL_FEATURES = [
"BMI",
"pCEA",
"Site_■■■■",
"Site_■■",
"cT Stage",
"Histology",
" Differentiation extent",
]
def _normalize_site(value: str) -> str:
x = str(value).strip().lower().replace("_", " ").replace("-", " ")
aliases = {
"right": "right",
"right colon": "right",
"right sided colon": "right",
"right-sided colon": "right",
"left": "left",
"left colon": "left",
"left sided colon": "left",
"left-sided colon": "left",
"rectum": "rectum",
"rectal": "rectum",
}
if x in aliases:
```

```
return aliases[x]
raise ValueError(
"primary_tumor_site must be one of: right-sided colon, left-sided colon, rectum."
)
def _normalize_ct_stage(value: Union[str, int]) -> int:
x = str(value).strip().lower().replace("stage", "").replace(" ", "")
mapping = {
"ct1": 0, "t1": 0, "1": 0,
"ct2": 1, "t2": 1, "2": 1,
"ct3": 2, "t3": 2, "3": 2,
"ct4": 3, "t4": 3, "4": 3,
}
if x in mapping:
return mapping[x]
raise ValueError("clinical_t_stage must be cT1, cT2, cT3, or cT4.")
def _normalize_histology(value: str) -> int:
x = str(value).strip().lower().replace("_", " ").replace("-", " ")
adenocarcinoma = {
"adenocarcinoma",
"conventional adenocarcinoma",
}
special = {
"mucinous adenocarcinoma",
"signet ring cell carcinoma",
"signet-ring cell carcinoma",
"mucinous adenocarcinoma/signet ring cell carcinoma",
"mucinous adenocarcinoma/signet-ring cell carcinoma",
"special",
"special histological type",
}
if x in adenocarcinoma:
return 0
if x in special:
return 1
raise ValueError(
"histological_type must be adenocarcinoma or "
"mucinous adenocarcinoma/signet-ring cell carcinoma."
)
def _normalize_differentiation(value: str) -> int:
x = str(value).strip().upper().replace(" ", "")
mapping = {
"G1": 0,
"G2": 1,
"G3": 2,
"G4": 2,
"G3/G4": 2,
"G3-4": 2,
}
if x in mapping:
return mapping[x]
raise ValueError("tumor_differentiation must be G1, G2, or G3/G4.")
def encode_patient(
bmi: float,
pcea: float,
primary_tumor_site: str,
clinical_t_stage: Union[str, int],
histological_type: str,
tumor_differentiation: str,
) -> pd.DataFrame:
"""Encode one patient's 6 predictors exactly as used by the fitted RF model."""
numeric_values = np.asarray([bmi, pcea], dtype=float)
if not np.all(np.isfinite(numeric_values)):
raise ValueError("BMI and pCEA must be nonmissing finite numeric values.")
```

```
if bmi <= 0:
raise ValueError("BMI must be greater than 0.")
if pcea < 0:
raise ValueError("pCEA must be nonnegative.")
site = _normalize_site(primary_tumor_site)
ct = _normalize_ct_stage(clinical_t_stage)
histology = _normalize_histology(histological_type)
differentiation = _normalize_differentiation(tumor_differentiation)
row = {
"BMI": float(bmi),
"pCEA": float(pcea),
"Site_[redacted]": int(site == "left"),
"Site_[redacted]": int(site == "rectum"),
"cT Stage": ct,
"Histology": histology,
" Differentiation extent": differentiation,
}
return pd.DataFrame([row], columns=MODEL_FEATURES)
def load_model(model_path: Union[str, Path, None] = None):
"""Load the supplied fitted random-forest model and verify feature compatibility."""
if model_path is None:
model_path = Path(__file__).resolve().with_name(MODEL_FILENAME)
else:
model_path = Path(model_path)
if not model_path.exists():
raise FileNotFoundError(f"Model file not found: {model_path}")
model = joblib.load(model_path)
if not hasattr(model, "predict_proba"):
raise TypeError("The loaded object does not provide predict_proba().")
stored_features = list(getattr(model, "feature_names_in_", []))
if stored_features and stored_features != MODEL_FEATURES:
raise ValueError(
"Feature names in the supplied model do not match the implementation. "
f"Expected {MODEL_FEATURES}; model contains {stored_features}."
)
if int(getattr(model, "n_features_in_", len(MODEL_FEATURES))) !=
len(MODEL_FEATURES):
raise ValueError("The supplied model does not contain the expected 7 encoded
features.")
return model
def predict_lnm(
bmi: float,
pcea: float,
primary_tumor_site: str,
clinical_t_stage: Union[str, int],
histological_type: str,
tumor_differentiation: str,
model_path: Union[str, Path, None] = None,
) -> dict:
"""Return the LNM probability and threshold-based model classification."""
X = encode_patient(
bmi=bmi,
pcea=pcea,
primary_tumor_site=primary_tumor_site,
clinical_t_stage=clinical_t_stage,
histological_type=histological_type,
tumor_differentiation=tumor_differentiation,
)
model = load_model(model_path)
classes = list(model.classes_)
if 1 not in classes:
raise ValueError("The supplied model does not contain class 1 (LNM-positive).")
positive_index = classes.index(1)
probability = float(model.predict_proba(X)[0, positive_index])
```

```
classification = "High risk" if probability >= RF_THRESHOLD else "Low risk"
return {
"predicted_lnm_probability": probability,
"threshold_full_precision": RF_THRESHOLD,
"threshold_rounded_3dp": round(RF_THRESHOLD, 3),
"model_classification": classification,
"note": (
"Research-stage model classification only; the threshold is training-derived "
"and is not a validated clinical treatment threshold."
),
}
def build_parser() -> argparse.ArgumentParser:
parser = argparse.ArgumentParser(
description=(
"Predict pathological LNM probability using the final 6-predictor "
"random-forest model."
)
)
parser.add_argument("--bmi", type=float, required=True, help="Body mass index
(kg/m^2).")
parser.add_argument("--pcea", type=float, required=True, help="Preoperative CEA
(ng/mL).")
parser.add_argument(
"--site",
required=True,
help='Primary tumor site: "right-sided colon", "left-sided colon", or "rectum".',
)
parser.add_argument(
"--ct",
required=True,
help='Clinical T stage: "cT1", "cT2", "cT3", or "cT4".',
)
parser.add_argument(
"--histology",
required=True,
help=(
'Histological type: "adenocarcinoma" or '
'"mucinous adenocarcinoma/signet-ring cell carcinoma".'
),
)
parser.add_argument(
"--differentiation",
required=True,
help='Tumor differentiation: "G1", "G2", or "G3/G4".',
)
parser.add_argument(
"--model",
default=None,
help=f"Optional path to the fitted model. Default: {MODEL_FILENAME} beside this
script.",
)
return parser
def main() -> None:
args = build_parser().parse_args()
result = predict_lnm(
bmi=args.bmi,
pcea=args.pcea,
primary_tumor_site=args.site,
clinical_t_stage=args.ct,
histological_type=args.histology,
tumor_differentiation=args.differentiation,
model_path=args.model,
)
print(json.dumps(result, indent=2, ensure_ascii=False))
```

```
if __name__ == "__main__":
main()
```

requirements.txt

```
scikit-learn==1.4.2
pandas>=2.0,<3.0
numpy>=1.24,<2.0
joblib>=1.2,<2.0
```
