# Supplementary material for: Preoperative Prediction of Lymph Node Metastasis in Colorectal Cancer Using Routinely Collected Clinical Data: Multicenter Retrospective Machine Learning Model Development and External Validation Study
Source: JMIR Cancer. 2026 Oct 7;12:e101162. doi: 10.2196/101162 (PMC13644950; doi:10.2196/101162)
Supplement: Supplementary file 2 [file cancer-v12-e101162-s001.docx]

# Table S1. Baseline characteristics of the training and internal test sets

| **Characteristic** | **Training set (n=753)** | **Internal test set (n=321)** | **Z or χ² value** | ***P* value** |
| --- | --- | --- | --- | --- |
| Age, years | 66.00 (59.00-73.00) | 66.00 (60.00-72.00) | -0.464 | .642 |
| **Sex** |  |  | 0.377 | .539 |
| Male | 481 (63.9%) | 198 (61.7%) |  |  |
| Female | 272 (36.1%) | 123 (38.3%) |  |  |
| **Ethnicity** |  |  |  | .572 |
| Han | 741 (98.4%) | 318 (99.1%) |  |  |
| Other | 12 (1.6%) | 3 (0.9%) |  |  |
| **Residence** |  |  | 0.356 | .551 |
| Urban | 543 (72.1%) | 225 (70.1%) |  |  |
| Rural | 210 (27.9%) | 96 (29.9%) |  |  |
| BMI, kg/m² | 23.94 (21.45-26.12) | 23.74 (20.96-26.35) | 0.860 | .390 |
| **Smoking status** |  |  |  | .574 |
| Never smoking | 610 (81.0%) | 264 (82.2%) |  |  |
| Current smoking | 132 (17.5%) | 55 (17.1%) |  |  |
| Former smoking | 11 (1.5%) | 2 (0.6%) |  |  |
| **Drinking status** |  |  |  | .687 |
| Never drinking | 649 (86.2%) | 277 (86.3%) |  |  |
| Current drinking | 94 (12.5%) | 42 (13.1%) |  |  |
| Former drinking | 10 (1.3%) | 2 (0.6%) |  |  |
| **Family history** |  |  |  | .371 |
| Yes | 3 (0.4%) | 3 (0.9%) |  |  |
| No | 750 (99.6%) | 318 (99.1%) |  |  |
| **Comorbidities** |  |  | 0.644 | .422 |
| Yes | 656 (87.1%) | 286 (89.1%) |  |  |
| No | 97 (12.9%) | 35 (10.9%) |  |  |
| **Bowel obstruction** |  |  | 0.016 | .899 |
| Yes | 152 (20.2%) | 63 (19.6%) |  |  |
| No | 601 (79.8%) | 258 (80.4%) |  |  |
| pCEA, ng/mL | 4.16 (2.30-9.28) | 4.33 (2.54-11.64) | -1.392 | .164 |
| pCA19-9, U/mL | 9.96 (5.06-19.40) | 9.93 (5.42-19.31) | -0.077 | .939 |
| Neutrophils, % | 63.60 (56.40-70.20) | 63.60 (55.90-70.50) | 0.139 | .889 |
| SII | 593.94 (407.13-994.73) | 608.14 (373.71-985.80) | 0.230 | .818 |
| AGR | 1.47 (1.28-1.66) | 1.44 (1.25-1.62) | 1.184 | .237 |
| **Primary tumor site** |  |  | 1.545 | .462 |
| Rectum | 312 (41.4%) | 142 (44.2%) |  |  |
| Left-sided colon | 231 (30.7%) | 101 (31.5%) |  |  |
| Right-sided colon | 210 (27.9%) | 78 (24.3%) |  |  |
| Maximum tumor diameter, cm | 3.50 (2.80-4.50) | 4.00 (3.00-5.00) | -1.679 | .092 |
| **Clinical T stage** |  |  | 5.168 | .160 |
| T1 | 50 (6.6%) | 15 (4.7%) |  |  |
| T2 | 148 (19.7%) | 75 (23.4%) |  |  |
| T3 | 498 (66.1%) | 215 (67.0%) |  |  |
| T4 | 57 (7.6%) | 16 (5.0%) |  |  |
| **Gross tumor type** |  |  | 0.939 | .332 |
| Ulcerative/infiltrative | 501 (66.5%) | 224 (69.8%) |  |  |
| Protruding | 252 (33.5%) | 97 (30.2%) |  |  |
| **Histological type** |  |  | 1.636 | .201 |
| Adenocarcinoma | 622 (82.6%) | 276 (86.0%) |  |  |
| Mucinous adenocarcinoma/signet-ring cell carcinoma | 131 (17.4%) | 45 (14.0%) |  |  |
| **Tumor differentiation** |  |  | 0.662 | .718 |
| G1 | 35 (4.6%) | 16 (5.0%) |  |  |
| G2 | 587 (78.0%) | 243 (75.7%) |  |  |
| G3/G4 | 131 (17.4%) | 62 (19.3%) |  |  |
| **Lymph node metastasis** |  |  | 0.000 | 1.000 |
| Yes | 278 (36.9%) | 118 (36.8%) |  |  |
| No | 475 (63.1%) | 203 (63.2%) |  |  |
| **Total number of lymph nodes examined** | 14.00 (12.00-17.00) | 15.00 (12.00-17.00) | -0.380 | .703 |

Values are presented as median (IQR) or n (%) unless otherwise indicated. Z tests were used for continuous variables with nonnormal distributions, and χ^2^ tests were used for categorical variables.

# Table S2. Missing data for candidate predictors before imputation in the training, internal test, and external validation sets.

| **Candidate predictor^a^** | **Training set (*N*=753), *n* (%)^b^** | **Internal test set (*N*=321), *n* (%)^b^** | **External validation set (*N*=1651), *n* (%)^b^** |
| --- | --- | --- | --- |
| **Demographic and general clinical variables** | | | |
| Age | 0 (0.00) | 0 (0.00) | 0 (0.00) |
| Sex | 0 (0.00) | 0 (0.00) | 0 (0.00) |
| Ethnicity | 0 (0.00) | 0 (0.00) | 2 (0.12) |
| Residence | 0 (0.00) | 0 (0.00) | 3 (0.18) |
| Smoking status | 2 (0.27) | 1 (0.31) | 12 (0.73) |
| Drinking status | 3 (0.40) | 1 (0.31) | 15 (0.91) |
| Family history | 4 (0.53) | 2 (0.62) | 18 (1.09) |
| Comorbidities | 3 (0.40) | 1 (0.31) | 12 (0.73) |
| Body mass index | 8 (1.06) | 3 (0.93) | 22 (1.33) |
| **Laboratory indices** | | | |
| Neutrophil percentage | 12 (1.59) | 4 (1.25) | 39 (2.36) |
| Systemic immune-inflammation index | 15 (1.99) | 6 (1.87) | 46 (2.79) |
| Albumin-to-globulin ratio | 14 (1.86) | 5 (1.56) | 42 (2.54) |
| **Tumor markers** | | | |
| Preoperative carcinoembryonic antigen | 28 (3.72) | 11 (3.43) | 78 (4.72) |
| Preoperative carbohydrate antigen 19-9 | 35 (4.65) | 14 (4.36) | 77 (4.66) |
| **Tumor-related variables** | | | |
| Clinical T stage | 7 (0.93) | 3 (0.93) | 19 (1.15) |
| Bowel obstruction | 4 (0.53) | 2 (0.62) | 18 (1.09) |
| Primary tumor site | 0 (0.00) | 0 (0.00) | 2 (0.12) |
| Maximum tumor diameter | 10 (1.33) | 4 (1.25) | 29 (1.76) |
| Gross tumor type | 8 (1.06) | 3 (0.93) | 26 (1.57) |
| Histological type | 5 (0.66) | 2 (0.62) | 15 (0.91) |
| Tumor differentiation | 9 (1.20) | 4 (1.25) | 20 (1.21) |

^a^Only prespecified preoperative candidate predictors are shown. Postoperative pathological lymph node metastasis status was the study outcome; patients without ascertainable outcome status were excluded before cohort formation, and outcome values were not imputed.
^b^Values are the number and percentage of observations with missing predictor data before imputation, presented as n (%). Percentages were calculated using the corresponding data set size (N) as the denominator. Missing predictor values were handled according to the prespecified procedure described in the Methods.

# Table S3. Agreement of histological type and tumor differentiation between preoperative biopsy and postoperative resection pathology.

**A. Cross-classification of histological type^a^**

| **Preoperative biopsy histological type** | **Postoperative adenocarcinoma (N=2120), n (row %)** | **Postoperative special histological type (N=583), n (row %)** | **Total, n** |
| --- | --- | --- | --- |
| Adenocarcinoma | 2057 (89.2) | 248 (10.8) | 2305 |
| Mucinous adenocarcinoma/signet-ring cell carcinoma | 63 (15.8) | 335 (84.2) | 398 |

**B. Cross-classification of tumor differentiation^b^**

| **Preoperative biopsy differentiation** | **Postoperative G1 (N=219), n (row %)** | **Postoperative G2 (N=1711), n (row %)** | **Postoperative G3/G4 (N=762), n (row %)** | **Total, n** |
| --- | --- | --- | --- | --- |
| G1 | 79 (73.1) | 27 (25.0) | 2 (1.9) | 108 |
| G2 | 138 (6.7) | 1580 (76.6) | 345 (16.7) | 2063 |
| G3/G4 | 2 (0.4) | 104 (20.0) | 415 (79.7) | 521 |

**C. Agreement statistics**

| **Statistic** | **Estimate** | **95% CI^c^** |
| --- | --- | --- |
| **Histological type** | | |
| Exact agreement, % | 88.5 | 87.2-89.6 |
| Missed special histological type among postoperative special types, % | 42.5 | 38.6-46.6 |
| Positive predictive value of a preoperative special-type diagnosis, % | 84.2 | 80.3-87.4 |
| Unweighted Cohen κ | 0.616 | 0.577-0.653 |
| **Tumor differentiation** | | |
| Exact agreement, % | 77.0 | 75.4-78.6 |
| Undergrading, % | 13.9 | 12.6-15.3 |
| Overgrading, % | 9.1 | 8.0-10.2 |
| Linearly weighted Cohen κ | 0.521 | 0.488-0.552 |

^a^Values are n (row %), with each preoperative biopsy category used as the denominator. Special histological type denotes mucinous adenocarcinoma or signet-ring cell carcinoma. A missed special histological type denotes adenocarcinoma on preoperative biopsy and a special histological type on postoperative resection pathology.

^b^Values are n (row %), with each preoperative biopsy category used as the denominator. G1 indicates well differentiated; G2, moderately differentiated; G3, poorly differentiated; and G4, undifferentiated. Undergrading denotes a lower grade on preoperative biopsy than on resection pathology; overgrading denotes a higher grade on preoperative biopsy.

^c^CI indicates confidence interval. Percentage CIs were calculated using Wilson intervals. CIs for Cohen κ were estimated using 20,000 multinomial bootstrap resamples.
Note: Agreement analyses were restricted to patients with nonmissing paired preoperative biopsy and postoperative resection pathology results (histological type, n=2703; tumor differentiation, n=2692). Imputed values were not used.

# Table S4. Univariable logistic regression analysis of factors associated with lymph node metastasis.

| **Variable** | **β** | **SE** | **OR (95% CI)** | ***z*** | ***P* value** |
| --- | --- | --- | --- | --- | --- |
| Age | -0.006 | 0.008 | 0.994 (0.979-1.009) | -0.781 | .435 |
| **Sex** | | | | | |
| Male | Ref |  |  |  |  |
| Female | 0.211 | 0.156 | 1.235 (0.909-1.677) | 1.348 | .178 |
| **Ethnicity** | | | | | |
| Han | Ref |  |  |  |  |
| Other | -0.571 | 0.671 | 0.565 (0.152-2.104) | -0.851 | .395 |
| **Residence** | | | | | |
| Urban | Ref |  |  |  |  |
| Rural | 0.042 | 0.168 | 1.042 (0.750-1.449) | 0.248 | .805 |
| BMI | 0.094 | 0.022 | 1.099 (1.052-1.147) | 4.277 | <.001 |
| **Smoking status** | | | | | |
| Never smoking | Ref |  |  |  |  |
| Current smoking | 0.120 | 0.197 | 1.128 (0.767-1.660) | 0.612 | .541 |
| Former smoking | -0.430 | 0.682 | 0.651 (0.171-2.478) | -0.630 | .529 |
| **Drinking status** | | | | | |
| Never drinking | Ref |  |  |  |  |
| Current drinking | -0.196 | 0.234 | 0.822 (0.520-1.300) | -0.837 | .402 |
| Former drinking | 0.108 | 0.651 | 1.114 (0.311-3.987) | 0.166 | .868 |
| **Family history** | | | | | |
| No | Ref |  |  |  |  |
| Yes | -0.158 | 1.227 | 0.854 (0.077-9.459) | -0.129 | .898 |
| **Comorbidities** | | | | | |
| No | Ref |  |  |  |  |
| Yes | -0.110 | 0.223 | 0.896 (0.578-1.387) | -0.493 | .622 |
| **Bowel obstruction** | | | | | |
| No | Ref |  |  |  |  |
| Yes | 0.376 | 0.184 | 1.457 (1.015-2.091) | 2.041 | .041 |
| pCEA | 0.006 | 0.002 | 1.006 (1.001-1.010) | 2.484 | .013 |
| pCA19-9 | 0.001 | 0.001 | 1.001 (1.000-1.002) | 1.551 | .121 |
| Neutrophil percentage | -0.003 | 0.007 | 0.997 (0.983-1.010) | -0.504 | .615 |
| SII | 0.000 | 0.000 | 1.000 (1.000-1.000) | -0.400 | .689 |
| AGR | -0.171 | 0.250 | 0.842 (0.516-1.374) | -0.686 | .493 |
| **Primary tumor site** | | | | | |
| Left-sided colon | Ref |  |  |  |  |
| Right-sided colon | 0.568 | 0.201 | 1.766 (1.190-2.620) | 2.822 | .005 |
| Rectum | 0.466 | 0.186 | 1.593 (1.107-2.292) | 2.509 | .012 |
| Maximum tumor diameter | 0.091 | 0.046 | 1.095 (1.001-1.199) | 1.978 | .048 |
| **Clinical T stage** | | | | | |
| T1 | Ref |  |  |  |  |
| T2 | -1.681 | 0.750 | 0.186 (0.043-0.810) | -2.241 | .025 |
| T3 | 2.109 | 0.480 | 8.238 (3.216-21.102) | 4.395 | <.001 |
| T4 | 2.444 | 0.542 | 11.520 (3.984-33.311) | 4.512 | <.001 |
| **Gross tumor type** | | | | | |
| Ulcerative/infiltrative | Ref |  |  |  |  |
| Protruding | -0.557 | 0.166 | 0.573 (0.413-0.794) | -3.349 | <.001 |
| **Histological type** | | | | | |
| Adenocarcinoma | Ref |  |  |  |  |
| Mucinous adenocarcinoma/signet-ring cell carcinoma | 0.788 | 0.194 | 2.199 (1.502-3.220) | 4.052 | <.001 |
| **Tumor differentiation** | | | | | |
| G1 | Ref |  |  |  |  |
| G2 | 2.105 | 0.733 | 8.208 (1.949-34.559) | 2.870 | .004 |
| G3/G4 | 3.286 | 0.750 | 26.730 (6.145-116.275) | 4.381 | <.001 |

**Abbreviations:** AGR, albumin-to-globulin ratio; BMI, body mass index; CI, confidence interval; OR, odds ratio; pCA19-9, preoperative carbohydrate antigen 19-9; pCEA, preoperative carcinoembryonic antigen; Ref, reference category; SE, standard error; SII, systemic immune-inflammation index; β, regression coefficient.
**Note:** Because β coefficients and SEs are displayed to 3 decimal places, z values for continuous predictors with very small coefficients may not be exactly reproducible from the rounded values shown.

**Table S5. Predictor preprocessing and encoding rules, hyperparameter search grids, and final settings for the candidate machine learning models.**

**A. Predictor preprocessing and encoding rules^a^**

| **Predictor type** | **Predictor** | **Preprocessing or encoding** | **Model input** |
| --- | --- | --- | --- |
| Continuous predictors | BMI; pCEA | Retained as continuous values; no standardization | Numeric |
| Nominal multicategory predictor | Primary tumor site | One-hot encoding | Right-sided colon as the reference; indicators for left-sided colon and rectum |
| Binary nominal predictor | Histological type | Binary encoding | Adenocarcinoma=0; mucinous adenocarcinoma/signet-ring cell carcinoma=1 |
| Ordinal predictor | Clinical T stage | Ordered integer encoding | cT1=0; cT2=1; cT3=2; cT4=3 |
| Ordinal predictor | Tumor differentiation | Ordered integer encoding | G1=0; G2=1; G3/G4=2 |

**B. Hyperparameter search grids and final settings for the candidate machine learning models^b^**

| Model | Hyperparameter search grid | Final setting |
| --- | --- | --- |
| Random forest | n_estimators: 100, 200; max_depth: 5, 8, 12, 16; min_samples_split: 2, 5, 10; min_samples_leaf: 1, 2, 4; class_weight: balanced, balanced_subsample, None | n_estimators=200; max_depth=5; min_samples_split=10; min_samples_leaf=4; class_weight=balanced |
| Support vector machine | C: 0.001, 0.01, 0.1, 1; kernel: linear, rbf | C=1; kernel=linear; probability=True |
| Logistic regression | Not applicable (not tuned) | penalty=l2; solver=lbfgs; C=1; max_iter=100; class_weight=None |
| Decision tree | max_depth: 10, 20; min_samples_split: 5, 10 | max_depth=10; min_samples_split=10 |
| XGBoost | n_estimators: 50, 100; max_depth: 3, 5, 10; learning_rate: 0.05, 0.1 | n_estimators=50; max_depth=3; learning_rate=0.1 |
| Naive Bayes | Not applicable (not tuned) | GaussianNB; priors=None; var_smoothing=1e-9 |
| LightGBM | n_estimators: 50, 100; max_depth: 3, 5, 10; learning_rate: 0.05, 0.1 | n_estimators=100; max_depth=3; learning_rate=0.05 |

a Preprocessing and encoding rules were fitted using the training data and then applied unchanged to the internal test and external validation data. Patients without ascertainable postoperative pathological lymph node metastasis status—the study outcome—were excluded before cohort formation, and outcome values were not imputed. Among eligible patients, missing continuous predictor values were handled using multiple imputation by chained equations with the R package mice, generating 3 imputed data sets with a maximum of 50 iterations per chain and a random seed of 123; missing categorical predictor values were imputed using the training-set mode.

b Hyperparameters for random forest, SVM, decision tree, XGBoost, and LightGBM were selected using GridSearchCV in the training set with 3-fold stratified cross-validation without shuffling and ROC AUC as the optimization criterion. Logistic regression and naive Bayes were fitted without hyperparameter tuning. Seeds were RF=39, SVM=42, DT=250, and XGBoost/LightGBM=10; SVM used probability=True. Unlisted parameters used package defaults.
Abbreviations: AUC, area under the receiver operating characteristic curve; BMI, body mass index; DT, decision tree; LightGBM, light gradient-boosting machine; pCEA, preoperative carcinoembryonic antigen; RF, random forest; ROC, receiver operating characteristic; SVM, support vector machine; XGBoost, extreme gradient boosting.

# Table S6. Pairwise DeLong comparisons of AUCs between the random forest model and the other candidate models in the internal test and external validation sets.

| **Data set** | **Comparator model** | **RF AUC** | **Comparator AUC** | **AUC difference (95% CI)^a^** | ***P* value^b^** | **Adjusted *P* value^c^** |
| --- | --- | --- | --- | --- | --- | --- |
| Internal test | Support vector machine | 0.803 | 0.790 | 0.013 (-0.019 to 0.046) | .43 | .86 |
| Internal test | Logistic regression | 0.803 | 0.778 | 0.024 (-0.014 to 0.062) | .22 | .66 |
| Internal test | Decision tree | 0.803 | 0.708 | 0.094 (0.048 to 0.141) | <.001 | <.001 |
| Internal test | XGBoost | 0.803 | 0.790 | 0.012 (-0.003 to 0.027) | .11 | .57 |
| Internal test | Naive Bayes | 0.803 | 0.809 | -0.006 (-0.033 to 0.021) | .67 | .86 |
| Internal test | LightGBM | 0.803 | 0.791 | 0.012 (-0.005 to 0.028) | .16 | .66 |
| External | Support vector machine | 0.776 | 0.751 | 0.025 (0.011 to 0.038) | <.001 | .001 |
| External | Logistic regression | 0.776 | 0.671 | 0.104 (0.086 to 0.122) | <.001 | <.001 |
| External | Decision tree | 0.776 | 0.675 | 0.100 (0.078 to 0.123) | <.001 | <.001 |
| External | XGBoost | 0.776 | 0.768 | 0.008 (0.001 to 0.014) | .03 | .06 |
| External | Naive Bayes | 0.776 | 0.734 | 0.041 (0.028 to 0.054) | <.001 | <.001 |
| External | LightGBM | 0.776 | 0.769 | 0.006 (-0.001 to 0.013) | .08 | .08 |

*Note.* AUC values and AUC differences are rounded to 3 decimal places.

^a^ AUC difference was calculated as RF AUC minus comparator AUC; positive values favor RF. The 95% CIs were derived from the DeLong standard errors.

^b^ Unadjusted 2-sided P values from the DeLong test.

^c^ P values were adjusted separately within each data set using the Holm method across the 6 RF-versus-comparator comparisons.

**Abbreviations:** AUC, area under the receiver operating characteristic curve; LightGBM, light gradient-boosting machine; RF, random forest; SVM, support vector machine; XGBoost, extreme gradient boosting.

# Table S7. Classification performance of the final random forest model at the training-derived Youden threshold and selected probability thresholds in the internal test and external validation sets

**A. Classification threshold and confusion matrix counts**

| **Data set** | **Sample size, n** | **LNM-positive, n (%)^b^** | **Threshold^a^** | **TP** | **FP** | **TN** | **FN** |
| --- | --- | --- | --- | --- | --- | --- | --- |
| Internal test | 321 | 118 (36.8) | 0.545 | 90 | 62 | 141 | 28 |
| External validation | 1651 | 619 (37.5) | 0.545 | 419 | 317 | 715 | 200 |

**B. Predictive values**

| **Data set** | **PPV (95% CI)^c^** | **NPV (95% CI)^c^** |
| --- | --- | --- |
| Internal test | 0.592 (0.511-0.670) | 0.834 (0.778-0.889) |
| External validation | 0.569 (0.534-0.605) | 0.781 (0.759-0.807) |

**C. Classification performance at selected probability thresholds**

| **Data set** | **Probability threshold^d^** | **Sensitivity** | **Specificity** | **PPV** | **NPV** | **FP, n** | **FN, n** |
| --- | --- | --- | --- | --- | --- | --- | --- |
| Internal test | 0.20 | 0.992 | 0.335 | 0.464 | 0.986 | 135 | 1 |
| Internal test | 0.40 | 0.966 | 0.522 | 0.540 | 0.964 | 97 | 4 |
| Internal test | 0.60 | 0.542 | 0.823 | 0.640 | 0.756 | 36 | 54 |
| Internal test | 0.80 | 0.042 | 0.995 | 0.833 | 0.641 | 1 | 113 |
| External validation | 0.20 | 1.000 | 0.326 | 0.471 | 1.000 | 696 | 0 |
| External validation | 0.40 | 0.922 | 0.481 | 0.516 | 0.912 | 536 | 48 |
| External validation | 0.60 | 0.511 | 0.827 | 0.638 | 0.738 | 179 | 303 |
| External validation | 0.80 | 0.039 | 0.991 | 0.727 | 0.632 | 9 | 595 |

*Note.*

^a^ The threshold of 0.545 was selected in the training set by maximizing the Youden index and was applied unchanged to the internal test and external validation sets.

^b^ Lymph node metastasis was treated as the positive outcome.

^c^ PPV and NPV values are point estimates (95% CIs) and are dependent on outcome prevalence.

^d^ Values are point estimates at selected probability thresholds. Thresholds of 0.20, 0.40, 0.60, and 0.80 were selected to illustrate threshold-dependent changes in classification performance and should not be interpreted as validated clinical decision thresholds.

**Abbreviations:** LNM, lymph node metastasis; TP, true positive; FP, false positive; TN, true negative; FN, false negative; PPV, positive predictive value; CI, confidence interval; NPV, negative predictive value.

# Table S8. Calibration performance of the candidate machine learning models in the training, internal test, and external validation sets

| **Model** | **Data set** | **Brier score (95% CI)^a^** | **Calibration intercept (95% CI)^a^** | **Calibration slope (95% CI)^a^** |
| --- | --- | --- | --- | --- |
| RF | Training | 0.165 (0.154 to 0.176) | −0.210 (−0.250 to −0.164) | 1.292 (1.183 to 1.374) |
| RF | Internal test | 0.179 (0.162 to 0.196) | −0.176 (−0.245 to −0.085) | 1.161 (0.886 to 1.358) |
| RF | External validation | 0.190 (0.183 to 0.197) | −0.120 (−0.158 to −0.077) | 1.061 (0.942 to 1.172) |
| SVM | Training | 0.177 (0.164 to 0.190) | 0.051 (−0.025 to 0.171) | 0.859 (0.486 to 1.065) |
| SVM | Internal test | 0.183 (0.164 to 0.199) | 0.084 (−0.052 to 0.207) | 0.723 (0.327 to 1.140) |
| SVM | External validation | 0.195 (0.184 to 0.203) | 0.050 (0.010 to 0.093) | 0.854 (0.728 to 0.957) |
| LR | Training | 0.202 (0.193 to 0.213) | −0.034 (−0.207 to 0.193) | 1.071 (0.416 to 1.498) |
| LR | Internal test | 0.200 (0.185 to 0.217) | 0.048 (−0.371 to 0.220) | 0.735 (0.261 to 2.045) |
| LR | External validation | 0.217 (0.210 to 0.225) | −0.014 (−0.143 to 0.204) | 0.920 (0.296 to 1.317) |
| DT | Training | 0.094 (0.082 to 0.104) | 0.000 (−0.072 to 0.078) | 1.000 (0.870 to 1.119) |
| DT | Internal test | 0.254 (0.215 to 0.293) | 0.331 (0.173 to 0.481) | 0.266 (0.027 to 0.512) |
| DT | External validation | 0.270 (0.252 to 0.288) | 0.321 (0.263 to 0.376) | 0.262 (0.164 to 0.361) |
| XGBoost | Training | 0.147 (0.135 to 0.158) | −0.101 (−0.145 to −0.048) | 1.266 (1.131 to 1.381) |
| XGBoost | Internal test | 0.175 (0.158 to 0.196) | −0.008 (−0.089 to 0.093) | 0.949 (0.681 to 1.183) |
| XGBoost | External validation | 0.187 (0.179 to 0.196) | 0.105 (0.054 to 0.158) | 0.737 (0.601 to 0.875) |
| NB | Training | 0.216 (0.199 to 0.233) | 0.185 (0.099 to 0.279) | 0.749 (0.444 to 0.973) |
| NB | Internal test | 0.218 (0.189 to 0.243) | 0.218 (0.059 to 0.374) | 0.855 (0.289 to 1.165) |
| NB | External validation | 0.234 (0.221 to 0.247) | 0.331 (0.233 to 0.416) | 0.280 (−0.049 to 0.617) |
| LightGBM | Training | 0.146 (0.136 to 0.157) | −0.095 (−0.143 to −0.044) | 1.260 (1.130 to 1.357) |
| LightGBM | Internal test | 0.175 (0.156 to 0.192) | −0.006 (−0.078 to 0.093) | 0.968 (0.697 to 1.128) |
| LightGBM | External validation | 0.187 (0.178 to 0.195) | 0.239 (0.085 to 0.275) | 0.342 (0.274 to 0.822) |

^a^ Data set sizes were 753 for the training set, 321 for the internal test set, and 1651 for the external validation set. Values are estimates (95% CIs). Ideal calibration corresponds to an intercept of 0 and a slope of 1; lower Brier scores indicate better overall probabilistic accuracy. Abbreviations: CI, confidence interval; DT, decision tree; LightGBM, light gradient boosting machine; LR, logistic regression; NB, naive Bayes; RF, random forest; SVM, support vector machine; XGBoost, extreme gradient boosting.

# Table S9. Decision curve analysis-derived threshold probability ranges for the candidate machine learning models in the training, internal test, and external validation sets

| **Model** | **Data set** | **Threshold probability range^a^** | **Range width^a^** |
| --- | --- | --- | --- |
| RF | Training | 0.05-0.80 | 0.75 |
| RF | Internal test | 0.05-0.80 | 0.75 |
| RF | External validation | 0.05-0.69 | 0.64 |
| SVM | Training | 0.05-0.69 | 0.64 |
| SVM | Internal test | 0.05-0.79 | 0.74 |
| SVM | External validation | 0.05-0.74 | 0.69 |
| LR | Training | 0.05-0.79 | 0.74 |
| LR | Internal test | 0.05-0.69 | 0.64 |
| LR | External validation | 0.05-0.58 | 0.53 |
| DT | Training | 0.05-0.80 | 0.75 |
| DT | Internal test | 0.05-0.54 | 0.49 |
| DT | External validation | 0.05-0.54 | 0.49 |
| XGBoost | Training | 0.05-0.80 | 0.75 |
| XGBoost | Internal test | 0.05-0.72 | 0.67 |
| XGBoost | External validation | 0.05-0.65 | 0.60 |
| NB | Training | 0.05-0.77 | 0.72 |
| NB | Internal test | 0.05-0.69 | 0.64 |
| NB | External validation | 0.05-0.53 | 0.48 |
| LightGBM | Training | 0.05-0.80 | 0.75 |
| LightGBM | Internal test | 0.05-0.74 | 0.69 |
| LightGBM | External validation | 0.05-0.65 | 0.60 |

^a^ Data set sizes were 753 for the training set, 321 for the internal test set, and 1651 for the external validation set. Threshold probability ranges were derived from the decision curve analysis over the prespecified interval of 0.05-0.80. Range width equals the upper minus the lower bound. A wider interval does not necessarily indicate a greater magnitude of net benefit; net benefit should be interpreted with the corresponding decision curves.
Abbreviations: DT, decision tree; LightGBM, light gradient boosting machine; LR, logistic regression; NB, naive Bayes; RF, random forest; SVM, support vector machine; XGBoost, extreme gradient boosting

# Table S10. Sensitivity analysis comparing random forest models using the 6 selected predictors, all candidate predictors, or clinical T stage alone in the internal test and external validation sets

**A. Discrimination and overall classification performance**

| **Data set** | **Predictor set** | **AUC (95% CI)^a^** | **Training-derived threshold^a^** | **Accuracy (95% CI)^a^** | **F1 score (95% CI)^a^** |
| --- | --- | --- | --- | --- | --- |
| Internal test | Selected 6 predictors | 0.803 (0.756 to 0.849) | 0.545 | 0.720 (0.670 to 0.769) | 0.667 (0.593 to 0.731) |
| Internal test | All candidate predictors | 0.833 (0.789 to 0.875) | 0.392 | 0.741 (0.695 to 0.788) | 0.680 (0.610 to 0.742) |
| Internal test | Clinical T stage alone | 0.712 (0.672 to 0.751) | 0.608 | 0.636 (0.583 to 0.685) | 0.665 (0.604 to 0.719) |
| External validation | Selected 6 predictors | 0.776 (0.755 to 0.797) | 0.545 | 0.687 (0.664 to 0.709) | 0.618 (0.588 to 0.649) |
| External validation | All candidate predictors | 0.787 (0.764 to 0.808) | 0.392 | 0.712 (0.689 to 0.733) | 0.652 (0.620 to 0.678) |
| External validation | Clinical T stage alone | 0.707 (0.691 to 0.724) | 0.608 | 0.622 (0.599 to 0.644) | 0.664 (0.639 to 0.687) |

**B. Classification performance at model-specific training-derived thresholds**

| **Data set** | **Predictor set** | **Training-derived threshold^a^** | **Sensitivity (95% CI)^a^** | **Specificity (95% CI)^a^** | **PPV (95% CI)^a^** | **NPV (95% CI)^a^** |
| --- | --- | --- | --- | --- | --- | --- |
| Internal test | Selected 6 predictors | 0.545 | 0.763 (0.677 to 0.834) | 0.695 (0.630 to 0.759) | 0.592 (0.511 to 0.670) | 0.834 (0.778 to 0.889) |
| Internal test | All candidate predictors | 0.392 | 0.746 (0.664 to 0.820) | 0.739 (0.680 to 0.797) | 0.624 (0.544 to 0.698) | 0.833 (0.780 to 0.886) |
| Internal test | Clinical T stage alone | 0.608 | 0.983 (0.957 to 1.000) | 0.433 (0.366 to 0.500) | 0.502 (0.437 to 0.567) | 0.978 (0.944 to 1.000) |
| External validation | Selected 6 predictors | 0.545 | 0.677 (0.644 to 0.716) | 0.693 (0.665 to 0.719) | 0.569 (0.534 to 0.605) | 0.781 (0.759 to 0.807) |
| External validation | All candidate predictors | 0.392 | 0.719 (0.684 to 0.753) | 0.707 (0.678 to 0.734) | 0.596 (0.559 to 0.631) | 0.808 (0.783 to 0.832) |
| External validation | Clinical T stage alone | 0.608 | 0.997 (0.992 to 1.000) | 0.397 (0.370 to 0.427) | 0.498 (0.470 to 0.525) | 0.995 (0.987 to 1.000) |

**C. Pairwise DeLong comparisons of AUC**

| **Data set** | **Comparison** | **Selected 6 AUC** | **Comparator AUC** | **AUC difference^b^** | **z value** | ***P value*** |
| --- | --- | --- | --- | --- | --- | --- |
| Internal test | Selected 6 vs clinical T stage alone | 0.803 | 0.712 | 0.091 | 3.849 | <.001 |
| External validation | Selected 6 vs clinical T stage alone | 0.776 | 0.707 | 0.069 | 6.566 | <.001 |
| Internal test | Selected 6 vs all candidate predictors | 0.803 | 0.833 | −0.030 | −1.448 | .148 |
| External validation | Selected 6 vs all candidate predictors | 0.776 | 0.787 | −0.011 | −1.224 | .221 |

**D. Calibration performance**

| **Data set** | **Predictor set** | **Calibration intercept (95% CI)^c^** | **Calibration slope (95% CI)^a^** | **O/E ratio (95% CI)^a^** | **Brier score (95% CI)^a^** |
| --- | --- | --- | --- | --- | --- |
| Internal test | Selected 6 predictors | −0.176 (−0.245 to −0.085) | 1.161 (0.886 to 1.358) | 0.806 (0.706 to 0.903) | 0.179 (0.162 to 0.196) |
| Internal test | All candidate predictors | −0.173 (−0.230 to −0.095) | 1.450 (1.161 to 1.656) | 0.978 (0.859 to 1.095) | 0.173 (0.158 to 0.188) |
| Internal test | Clinical T stage alone | −0.065 (−0.088 to −0.037) | 0.899 (0.768 to 1.023) | 0.804 (0.698 to 0.910) | 0.196 (0.177 to 0.214) |
| External validation | Selected 6 predictors | −0.120 (−0.158 to −0.077) | 1.061 (0.942 to 1.172) | 0.803 (0.758 to 0.848) | 0.190 (0.183 to 0.197) |
| External validation | All candidate predictors | −0.134 (−0.166 to −0.098) | 1.381 (1.268 to 1.493) | 1.005 (0.951 to 1.062) | 0.185 (0.177 to 0.192) |
| External validation | Clinical T stage alone | −0.078 (−0.085 to −0.071) | 0.915 (0.863 to 0.968) | 0.787 (0.743 to 0.830) | 0.200 (0.193 to 0.207) |

**E. Decision curve analysis-derived threshold probability ranges**

| **Data set** | **Predictor set** | **Lower threshold** | **Upper threshold** | **Range width^d^** |
| --- | --- | --- | --- | --- |
| Internal test | Selected 6 predictors | 0.05 | 0.80 | 0.75 |
| External validation | Selected 6 predictors | 0.05 | 0.69 | 0.64 |
| Internal test | All candidate predictors | 0.05 | 0.78 | 0.73 |
| External validation | All candidate predictors | 0.05 | 0.79 | 0.74 |
| Internal test | Clinical T stage alone | 0.05 | 0.50 | 0.45 |
| External validation | Clinical T stage alone | 0.05 | 0.49 | 0.44 |

^a^ Values are estimates (95% CIs). Accuracy, sensitivity, specificity, PPV, NPV, and F1 score were calculated using model-specific Youden thresholds derived from the training set and applied unchanged to the internal test and external validation sets. The thresholds were 0.545 for the selected 6-predictor model, 0.392 for the all-candidate-predictor model, and 0.608 for the clinical T stage-only model.

^b^ AUC difference was calculated as the AUC of the selected 6-predictor model minus that of the comparator model.

^c^ Ideal calibration corresponds to an intercept of 0, a slope of 1, and an O/E ratio of 1; lower Brier scores indicate better overall probabilistic accuracy.

^d^ Decision curve analysis was conducted over the prespecified threshold probability interval of 0.05-0.80. Reported ranges were restricted to this interval. Range width equals the upper minus the lower threshold probability. A wider range does not necessarily indicate a greater magnitude of net benefit.

Abbreviations: AUC, area under the receiver operating characteristic curve; CI, confidence interval; NPV, negative predictive value; O/E, observed-to-expected; PPV, positive predictive value.

**Figures**

# Figure S1. Global SHAP feature importance of the final random forest model.

Feature importance was calculated in the training set using mean absolute SHAP values. Primary tumor site was one-hot encoded as “Left-sided colon” and “Rectum,” with right-sided colon serving as the reference category.

**Abbreviations:** BMI, body mass index; CEA, carcinoembryonic antigen; SHAP, Shapley additive explanations.

# **Figure S2.** Individual-level SHAP explanation for a representative case.

Positive SHAP values increased the predicted probability of lymph node metastasis, whereas negative SHAP values decreased it. Primary tumor site was one-hot encoded, with right-sided colon serving as the reference category. Abbreviations: BMI, body mass index; CEA, carcinoembryonic antigen; LNM, lymph node metastasis; SHAP, Shapley additive explanations.

# **Figure S3.** SHAP dependence plots for all encoded features in the final random forest model.

Each point represents one patient. Positive and negative SHAP values indicate contributions toward higher and lower predicted risks of lymph node metastasis, respectively. Red lines represent LOWESS trends for continuous predictors. Primary tumor site was one-hot encoded using right-sided colon as the reference category. The preoperative carcinoembryonic antigen axis is displayed on a logarithmic scale. SHAP, SHapley Additive exPlanations; LOWESS, locally weighted scatterplot smoothing.

# **Figure S4.** Sensitivity analysis of random forest models based on different predictor sets in the internal test and external validation sets.

The top, middle, and bottom rows show receiver operating characteristic curves, calibration curves, and decision curve analyses, respectively. Abbreviations: AUC, area under the receiver operating characteristic curve; DCA, decision curve analysis; RF, random forest; ROC, receiver operating characteristic.
